# Neuregulin 1 Type II-ErbB Signaling Promotes Cell Divisions Generating Neurons from Neural Progenitor Cells in the Developing Zebrafish Brain

**DOI:** 10.1371/journal.pone.0127360

**Published:** 2015-05-22

**Authors:** Tomomi Sato, Fuminori Sato, Aosa Kamezaki, Kazuya Sakaguchi, Ryoma Tanigome, Koichi Kawakami, Atsuko Sehara-Fujisawa

**Affiliations:** 1 Department of Growth Regulation, Institute for Frontier Medical Sciences, Kyoto University, Sakyo-ku, Kyoto, Japan; 2 Laboratory of Molecular Cell Biology and Development, Graduate School of Biostudies, Kyoto University, Sakyo-ku, Kyoto, Japan; 3 Division of Molecular and Developmental Biology, National Institute of Genetics, Mishima-shi, Shizuoka, Japan; Institut Curie, FRANCE

## Abstract

Post-mitotic neurons are generated from neural progenitor cells (NPCs) at the expense of their proliferation. Molecular and cellular mechanisms that regulate neuron production temporally and spatially should impact on the size and shape of the brain. While transcription factors such as *neurogenin1* (*neurog1*) and *neurod* govern progression of neurogenesis as cell-intrinsic mechanisms, recent studies show regulatory roles of several cell-extrinsic or intercellular signaling molecules including Notch, FGF and Wnt in production of neurons/neural progenitor cells from neural stem cells/radial glial cells (NSCs/RGCs) in the ventricular zone (VZ). However, it remains elusive how production of post-mitotic neurons from neural progenitor cells is regulated in the sub-ventricular zone (SVZ). Here we show that newborn neurons accumulate in the basal-to-apical direction in the optic tectum (OT) of zebrafish embryos. While neural progenitor cells are amplified by mitoses in the apical ventricular zone, neurons are exclusively produced through mitoses of neural progenitor cells in the sub-basal zone, later in the sub-ventricular zone, and accumulate apically onto older neurons. This neurogenesis depends on Neuregulin 1 type II (NRG1-II)–ErbB signaling. Treatment with an ErbB inhibitor, AG1478 impairs mitoses in the sub-ventricular zone of the optic tectum. Removal of AG1478 resumes sub-ventricular mitoses without precedent mitoses in the apical ventricular zone prior to basal-to-apical accumulation of neurons, suggesting critical roles of ErbB signaling in mitoses for post-mitotic neuron production. Knockdown of NRG1-II impairs both mitoses in the sub-basal/sub-ventricular zone and the ventricular zone. Injection of soluble human NRG1 into the developing brain ameliorates neurogenesis of NRG1-II-knockdown embryos, suggesting a conserved role of NRG1 as a cell-extrinsic signal. From these results, we propose that NRG1-ErbB signaling stimulates cell divisions generating neurons from neural progenitor cells in the developing vertebrate brain.

## Introduction

Generation of neurons is an initial step to obtain higher brain functions during development [1]. In development of the mammalian brain, post-mitotic neurons are basically generated through two steps; first, neural stem cells/radial glial cells (NSCs/RGCs) produce neural progenitor cells (NPCs; intermediate/basal progenitor cells) by asymmetric cell divisions in the apical ventricular zone (VZ), and second, neural progenitor cells produce post-mitotic neurons by symmetric cell divisions in the sub-ventricular zone (SVZ) [2,3]. Newborn neurons migrate along radial fibers to form layers in an inside-out manner [4,5]. Neural progenitor cells proliferate in the sub-ventricular zone and produce post-mitotic neurons at the expense of their proliferation. Thus, the balance between proliferation and differentiation of neural progenitor cells should impact on the pool size of neural progenitor cells and the total number of neurons that contribute to the size and shape of the brain [1,3]. It is well established that expression of basic helix-loop-helix (bHLH) transcription factors such as *hes1*, *neurogenin1* (*neurog1*) and *neurod* determine proliferation of radial glial cells, generation of neural progenitor cells and differentiation of neurons, respectively, and therefore govern progression of neurogenesis as cell-intrinsic mechanisms [6,7]. In addition, recent studies reveal several intercellular signaling molecules including Notch, FGF, and Wnt that play regulatory roles in generation of neurons/neural progenitor cells from neural stem/radial glial cells as cell-extrinsic mechanisms in the ventricular zone [3,4]. However, it remains elusive how generation of neurons from neural progenitor cells is regulated in the sub-ventricular zone, in particular, whether the process generating neurons from neural progenitor cells requires cell-extrinsic mechanisms or it merely depends on cell-intrinsic mechanisms.

Neuregulin 1 (NRG1)-ErbB signaling is known to be a multi-potent regulator of cellular behaviors and functions in the nervous systems including proliferation, differentiation and migration of neural stem/progenitor cells and glial cells as well as myelination, synaptogenesis, and synaptic plasticity [8–10]. Also, the *nrg1* and *erbb4* genes are linked as susceptibility loci for a mental disorder, schizophrenia [9,11–13]. NRG1 is a member of epidermal growth factor (EGF) ligand family, and binds to ErbB3 and ErbB4 receptor tyrosine kinases [8,9]. NRG1 has multiple isoforms by alternative splicing that are classified into 6 types (type I-VI) according to the N-terminal domains in mammals [9]. Thus, various roles of NRG1-ErbB signaling would be, in part, due to multiple isoforms of NRG1. Indeed, different isoforms of NRG1 likely modulate synaptic plasticity; normal sensory-motor gating and short-term memory requires NRG1 type III [14], while a proper expression level of NRG1 type I is prerequisite for normal synaptic transmissions and mouse behaviors [15]. Myelination in both peripheral and central nervous systems is mainly regulated by NRG1 type III [16,17]. On the other hand, previous reports using *in vitro* cell culture systems suggest that NRG1 plays roles in establishment and maintenance of radial glial cells [18], neuronal migration along radial glial fibers [19,20], and proliferation of neural stem cells and/or neural progenitor cells [21]. However, little is known about which isoforms of NRG1 are involved in neurogenesis *in vivo*.

The optic tectum (OT, the mammalian superior colliculus) is a center of visuomotor behaviors in the midbrain, showing a layered structure that receives visual inputs from the retina in the superficial layers and sends motor outputs from the deeper layers to the hindbrain [22,23]. The optic tectum occupies the most expanded area in the zebrafish brain [24], like the neocortex in the mammalian brain, although the neuroanatomical structures and functions are quite different between them. Thus, the zebrafish optic tectum is one of the best model systems to investigate not only fundamental molecular mechanisms for generation of neurons *in vivo*, but also evaluate functional consequences of those mechanisms in neural circuit formation and visuomotor behaviors during development [25–27]. In the developing optic tectum, Sonic hedgehog (Shh)-Gli signaling has been reported to control neural stem/radial glial cell divisions in zebrafish [28], which is consistent with a role of Shh signaling in regulation of proliferative versus neurogenic cell divisions of neural stem/radial glial cells in the ventricular zone of the developing neocortex [29]. In contrast, the mechanisms for regulating cell divisions of neural progenitor cells to generate neurons in the sub-ventricular zone are poorly understood.

Here, we reveal that NRG1 type II (NRG1-II)—ErbB signaling is required for the generation of post-mitotic neurons from neural progenitor cells as a cell-extrinsic signal. In the developing optic tectum of zebrafish embryos, post-mitotic neurons are produced through mitoses of neural progenitor cells initially in the sub-basal zone, later in the sub-ventricular zone, and accumulate in the basal-to-apical (outside-in) direction. Neural progenitor cells proliferate in the ventricular zone through interkinetic nuclear migration. Treatment with AG1478 inhibits generation of post-mitotic neurons and mitoses in the sub-ventricular zone. The suppressed sub-ventricular mitoses are restored after removal of AG1478 prior to recovery of post-mitotic neuron generation, suggesting requirement of ErbB signaling in sub-ventricular mitoses for the production of post-mitotic neurons. Knockdown of NRG1-II impairs generation of post-mitotic neurons and both mitoses in the ventricular zone and sub-basal/sub-ventricular zone. The impaired neurogenesis in NRG1-II-knockdown embryos is ameliorated by injection of soluble human NRG1 into the ventricle of the developing brain, suggesting a conserved role of NRG1-ErbB signaling in neurogenesis as a cell-extrinsic signal. From these results, we propose a model in which NRG1-ErbB signaling stimulates cell divisions for generation of post-mitotic neurons by promoting neurogenic competence of neural progenitor cells in the vertebrate brain during development.

## Materials and Methods

### Ethics statement

All vertebrate animal experiments were performed at the facilities of Institute for Frontier Medical Sciences, Kyoto University in accordance with the Regulation on Animal Experimentation at Kyoto University. This study was approved by the Animal Experimentation Committee of Kyoto University (J-15). All efforts were made to minimize suffering during experimental procedures.

### Zebrafish husbandry and transgenic lines

Zebrafish were maintained at 28°C under 14 hours of light/10 hours of dark cycles and kept as described previously [30]. Embryos from the transgenic zebrafish were used as follows: Tg(*pou4f1-hsp70l*:*GFP*) (previously Tg(*brn3a-hsp70*:*GFP*)) [31] to visualize the development of post-mitotic neurons in the optic tectum; Tg(*elavl3*:*Kaede*) (previously Tg(*HuC*:*Kaede*)) [32] to segregate the timing of post-mitotic neuron generation; TgBAC(*neurod*:*EGFP*) [33] to visualize differentiated neurons; Tg(*-8*.*6neurog1*:*nRFP*) [34] to visualize neural progenitor cells; Tg(*neurog1*:*lRl-GFP*) [35] to observe neural progenitor cells; and SAGFF(LF)81C [36] to stochastically label cells in the optic tectum by co-injection with *UAS*:*membGFP* and *UAS*:*mCherry*.

### Morpholino injections

Morpholino oligonucleotides (MO) were diluted in 1× Danieau buffer (58 mM NaCl, 0.7 mM KCl, 0.4 mM MgSO_4_, 0.6 mM Ca(NO_3_)_2_, 5.0 mM HEPES pH 7.6) for injection at concentrations shown below. Approximately 1–5 nl of MO solutions was injected into 1 to 4-cell-stage embryos. MO-injected embryos with severe abnormalities in gross morphology were removed at 24 hpf. MO sequences and the concentrations for injections are shown below: MO^*erbb4atg*^ (5’-CGAACCGGCCACATTTCTGCTTATT-3’) at 3.35 ng/nl, MO^*erbb4KD*^ (5’-TACCCTGCCGAAAAAGTCCAAAAAC-3’) at 0.42 ng/nl, MO^*erbb4KD5m*^ (5’-TACCGTCCCGAATAACTCGAAAAAC-3’) at 0.42 ng/nl, MO^*nrg1EGF*^ (5’-TGCTGGTGGCTGCTGCACAGAGGAA-3’) [37] at 0.86 ng/nl, MO^*nrg1TM*^ (5’-TCTGGAAGAGAGGACAGACTGCTTT-3’) at 2.56 ng/nl, MO^*nrg1TM5m*^ (5’-TCAGCAAGACAGGACACACTCCTTT-3’) at 2.52 ng/nl, MO^*nrg1IIatg*^ (5’-TGCGTCCTGGCAGAATCGCCATCTC-3’) at 2.52 ng/nl, MO^*nrg1IIatg5m*^ (5’-TGCCTCGTGGCACAATCCCCATATC- 3’) at 2.50 ng/nl, MO^*nrg1IIE1*^ (5’-ACACTCTTAAAGGGTCTTACCGCAA-3’) at 8.41 ng/nl, MO^*nrg1IIE15m*^ (5’-ACAGTGTTAAACGGTCTTAGCCCAA-3’) at 8.45 ng/nl.

### Morpholino specificity

An expression plasmid *CMV_nrg1II-EGFP* was constructed by inserting a sequence of *nrg1-II* (aattcatccagctgaacgcaaggaccgcgcgcggagatggcgattctgccaggacgcagc) including non-coding and coding region of the first exon into the ATG initiation codon of EGFP, and the *nrg1II-EGFP* fragment was inserted between Hind III and Eco RI sites of pcDNA3.1(+) expression vector (Life technologies). The *CMV_nrg1II-EGFP* plasmid at a concentration of 20 ng/μl was co-injected with MO^*nrg1IIatg*^ or the control MO^*nrg1IIatg5m*^ into 1 to 4-cell-stage embryos, and GFP-expressing embryos were counted under a fluorescent stereomicrocope Leica MZ FL III at 25–27 hpf. Images of representative co-injected embryos were captured at about 76 hpf using a fluorescent microscope BZ-9000 (Keyence).

### Whole-mount *in situ* hybridization

Whole-mount *in situ* hybridization (WISH) was performed according to standard protocols [30]. cDNA clones of *her6* (accession number BM259629), *neurogenin1* (NM_131041), *neurod* (AF036148), *erbb4* (EF190457), full-length *type II nrg1* (FJ593488) were used to prepare riboprobes. Primers for amplification of the template cDNA were designed as follows: *her6* (5’- AAATGACCGCTGCCCTAAACACAG -3’, 5’-CATTTCAAATGTCATTTATTTGTCTTCCAA-3’), *neurogenin1* (5’- ATGGAGATCGTATACTCCGATATGG -3’, 5’- TTAATAGATGCTAGGCACGAAGTTGC -3’), *neurod* (5’- ATGACGAGGTCATACAGCGAGGAAAG -3’, 5’- TCACGAGTCGTGAAATATCGCGTTCAAC -3’), *erbb4* (5’- AGACTCAGACGCTGGACTGTCAATGCC -3’, 5’- GCATGAGCTGAGTGACCAGCTGAATAGTGGG -3’), *nrg1* (5’-AAGGACCGCGCGCGGAGATGGCGATTC-3’, T3 primer). Digoxigenin-labelled RNA probes were prepared using a DIG RNA labeling kit (Roche Applied Science). Cryosections were prepared after WISH.

### Immunofluorescent staining

Cryostat sectioning and immunofluorescent staining of embryos were performed according to standard protocols [30]. We used the following antibodies in this study: anti-activated caspase-3 (rabbit, 1:500 dilution; BD Pharmingen), anti-phospho-histone H3 (pH3; rabbit, 1:500; Cell Signaling) and anti-phsopho-HER4 pTyr1188 (rabbit, 1:25 dilution; Merck Millipore).

### Microscopy

Images were captured using a Leica TCS SP5 or SP8 confocal microscope equipped with Leica HyD using HC PL APO CS2 20×/NA 0.75, 40×/NA 1.30 and 63×/NA 1.40 objectives, and an Olympus FluoView FV1000D confocal microscope using UPLSAPO 20× NA 0.75 and 40× 2 NA 0.95 objectives.

### Photoconversion

Tg(*elavl3*:*Kaede*) embryos were protected from light before and after photoconversion [32]. For photoconversion, ultraviolet light (UV) was illuminated to the entire brain of embryos viewed under an Olympus IX81 microscope with a UPLSAPO 10× 2 NA 0.40 objective for approximately 60 s per embryo.

### ErbB inhibitor treatment

5 mM stock solutions of ErbB inhibitor AG1478 (Merck Millipore) prepared in DMSO were used at a final concentration of 30 μM in 2% DMSO. Control embryos were treated with 30 μM AG43 (Merck Millipore) or 2% DMSO. Embryos were removed from chorions, and immersed in water containing the inhibitor at 25–28 hpf. The inhibitors were washed out at 45 hpf for live imaging, otherwise embryos were fixed in 4% paraformaldehyde.

### Timelapse imaging

For stochastic labeling of neural progenitor cells, a mixture of two plasmids, *UAS*:*membGFP* and *UAS*:*mCherry* (25 ng/μl each) was injected into 1 to 4-cell-stage embryos from the SAGFF(LF)81C gene-trap line [23,36]. Injected embryos were treated with AG1478 as described before. The treated embryos with labelled cells in the optic tectum were selected under Leica M205 C fluorescent stereomicroscopy, and mounted in 1.5% low-melting-point agarose (Nacalai tesque) in a 35-mm glass-bottom dish (MatTek). Time-lapse images were captured under Olympus FV1000D confocal microscope with UPLSAPO 40x 2 NA 0.95 objective, at 10–20 min intervals for 15 hours.

### Recombinant protein injections

Human NRG1 recombinant proteins (BD Biosciences) were diluted at 1.0 mg/ml with PBS containing 20 mg/ml BSA, and pressure-injected into the hindbrain ventricles of MO-injected embryos mounted in 1.5% low-melting-point agarose at 48 hpf. The successful injections were confirmed by fluorescent signals from the 0.125% tetramethyl rhodamine dextran in the injection solution.

### Statistical analyses

GFP intensity in the optic tectum was used as an indicator of neurogenesis that represents both the number of neurons and the differentiation. GFP intensity was quantified by a value within ± 1.0–1.5 s.d. in the optic tectum. GFP intensity, sum of areas or volumes of pErbB4 and the number of pH3-positive cells in the optic tectum were quantified using Volocity (PerkinElmer). Relative values were calculated as ratios to the controls. For determination of statistical significance between two groups, either unpaired Student’s t test (equal variances) or t test with Welch’s correction (unequal variances) was used through Prism (GraphPad). To compare three or more groups, one-way ANOVA with Bonferroni’s post hoc test was used for multiple comparisons. Probability values (*P* values) < 0.05 were considered to be statistically significant (*P < 0.05, **P < 0.01. ***P < 0.001, ****P < 0.0001). Values indicated are means ± s.e.m.

## Results

### A landscape of neurogenesis in the developing optic tectum

**Fig 1:**
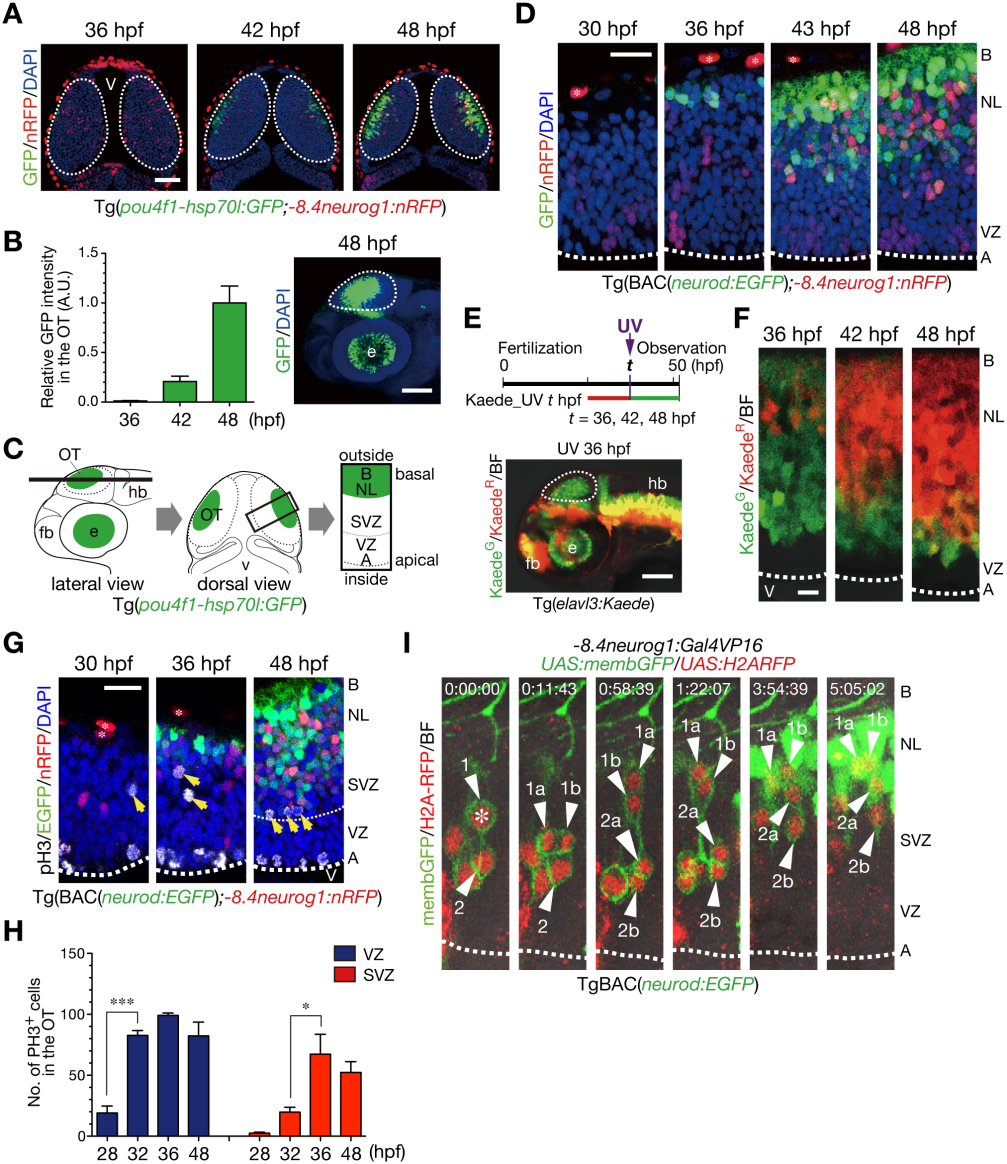
Generation of neurons is spatially and temporally regulated in the optic tectum of zebrafish embryos. **A**. Representative Tg(*pou4f1-hsp70l*:*GFP;-8*.*4neurog1*:*nRFP*) embryos in a dorsal view. GFP-positive neurons gradually expand from the outside (basal) to the inside (apical) region. The *-8*.*4neurog1*:nRFP is weakly expressed in a subset of NPCs, and highly expressed in pigment cells on the surface of the brain. Dotted circle, optic tectum (OT); v, ventricle. Scale bars, 50 μm. **B**. Quantification of *pou4f1-hsp70l*:GFP intensity in the OT (mean ± s.e.m.; n = 3, 5, 5 for 36, 42, 48 hpf, respectively). A.U., arbitrary units. Representative Tg(*pou4f1-hsp70l*:*GFP*) embryos at 48 hpf in a lateral view (right). Dotted circle, OT; e, eye. Scale bar, 100 μm. **C**. Schematics of confocal images from lateral (left) and dorsal (middle) views of the OT, and a image at a higher magnification from a dorsal view (right). Dotted circle, OT; fb, forebrain; hb, hindbrain; A, apical; B, basal; NL, neuronal layer; SVZ, sub-ventricular zone; VZ, ventricular zone. **D**. The OT of Tg(BAC(*neurod*:*GFP*);*-8*.*4neurog1*:*nRFP*) embryos in dorsal views. Dotted line, apical surface of the VZ; asterisk, pigment cell in the skin. Scale bar, 20 μm. **E**. (top) A timeline of experiments for UV irradiation. Embryos were subjected to UV irradiation at 36, 42 and 48 hpf. Neurons expressing Kaede were fluorescently labeled in red by the irradiation and neurons generated after the irradiation were labeled in green. (bottom) A lateral view of Tg(*elavl3*:*Kaede*) embryos irradiated with UV light at 36 hpf. Dotted circle, OT. Scale bar, 50 μm. **F**. Accumulation of older post-mitotic neurons (Kaede^R^) from the basal to apical regions of the OT in Tg(*elavl3*:*Kaede*). Dotted line, apical surface of the VZ. Scale bar, 10 μm. **G**. pH3-positive mitotic cells in the OT of Tg(BAC(*neurod*:*GFP*);*-8*.*4neurog1*:*nRFP*). Yellow arrows, pH3-positive cells in the sub-basal zone/SVZ. Thick dotted line, apical surface of the VZ; thin dotted line, approximate boundary between the VZ and the SVZ. **H**. Quantification of the number of pH3-positive cells in the VZ (blue) and the SVZ (red) of the developing OT (mean ± s.e.m.; *P < 0.05, ***P < 0.001; n = 3 per group). **I**. Time-lapse imaging of NPCs stochastically labeled by co-injection of *-8*.*4neurog1*:*Gal4VP16*/*UAS*:*memb*:*GFP*/*UAS*:*H2ARFP* plasmids into TgBAC(*neurod*:*EGFP*) embryos. Two NPCs (1, 2) underwent mitoses in the SVZ (asterisk) to produce two daughter cells (a, b) that ultimately differentiated into *neurod*:GFP-expressing post-mitotic neurons in the neuronal layer (NL; 1a, 1b, 2a). See also S1 Movie.

To obtain a landscape of neurogenesis during early stages of development in the vertebrate brain, we observed Tg(*pou4f1*(previously referred to as *brn3a*)*-hsp70l*: *GFP;-8*.*4neurog1*:*nRFP*) double-transgenic zebrafish embryos in which GFP and RFP with a nuclear localization signal (nRFP) were expressed in post-mitotic neurons and in some of neural progenitor cells in the optic tectum, respectively [26,30]. *pou4f1-hsp70l*:GFP-expressing neurons initially emerged in the most outside (basal) region of the optic tectum at 36 hours post fertilization (hpf), and neuronal differentiation proceeded toward the inside (apical) region of the optic tectum (Fig 1A and 1C). Those neurons were exponentially increased in number until 48 hpf (Fig 1B). Development of post-mitotic neurons depends on the expression of a transcription factor NeuroD [6]. Consistently, in Tg(BAC(*neurod*:*EGFP*);*-8*.*4neurog1*:*nRFP*) embryos [33], *neurod*:EGFP-positive cells initially appeared in the most basal region and then expanded apically, which is in contrast to apical-to-basal distribution of *-8*.*4neurog1*:nRFP-positive neural progenitor cells (Fig 1D). To examine whether the first-born neurons are located in the most basal regions and younger neurons are positioned apically onto older ones, living embryos of Tg(*elavl3*(previously referred to as *huC*):*Kaede*) transgenic line were subjected to irradiating ultraviolet (UV) light between 36 and 48 hpf to convert the fluorescence of Kaede expressed in neurons from green to red, which enabled us to distinguish between younger and older neurons at each time point of the irradiation [32,38] (Fig 1E). As was expected, younger neurons showing green fluorescence were essentially located more apically to the layer of older neurons showing red fluorescence, indicating the basal-to-apical accumulation, or outside-in generation, of neurons in the early phase of neurogenesis in the optic tectum (Fig 1F). Thus, generation of neurons is a process tightly regulated temporally and spatially.

### Mitoses of neural progenitor cells in the sub-ventricular zone produce post-mitotic neurons

Post-mitotic neurons are produced through mitoses of neural progenitor cells [39–41]. The question is when and where those mitoses of neural progenitor cells occur in the optic tectum of zebrafish embryos. In Tg(BAC(*neurod*:*EGFP);-8*.*4neurog1*:*nRFP*) embryos, phospho-histone H3 (pH3)-positive mitotic cells were distributed in two distinct layers in the optic tectum, one in the apical surface of the ventricular zone and the other in the sub-basal zone, that is, in the proximity of the neuronal layer, later in the sub-ventricular zone at 48 hpf (Fig 1G). The number of mitotic cells in both the layers peaked at around 36 hpf. Mitotic cells in the sub-ventricular zone were significantly increased between 32 hpf and 36 hpf (Fig 1H), which roughly corresponds to a period of emergence of post-mitotic neurons (Fig 1A and 1D). This implies that mitoses in the sub-basal/sub-ventricular zone contribute to the production of post-mitotic neurons. To monitor mitoses of neural progenitor cells in living embryos, three plasmids containing constructs *-8*.*4neurog1*:*Gal4VP16*, *UAS*:*membGFP* and *UAS*:*H2A-RFP* were co-injected into fertilized eggs of TgBAC(*neurod*:*EGFP*) transgenic line. This enabled us to stochastically double-label neural progenitor cells with membrane-GFP (membGFP) and H2A-RFP by expression of Gal4VP16 under the control of a *neurog1* promoter, which visualized clustered cells derived from single neural progenitor cells (Fig 1I, S1A Fig, S1 and S2 Movies). Live imaging of these embryos from 30 hpf to 45 hpf revealed that mitoses of neural progenitor cells in the sub-ventricular zone gave rise to *neurod*:GFP-positive post-mitotic neurons (Fig 1I and S1 Movie), while mitoses of neural progenitor cells in the ventricular zone gave rise to clonally aligned neural progenitor cells after interkinetic nuclear migrations (S1A Fig and S2 Movie). Indeed, in the developing optic tectum of Tg(BAC(*neurod*:*EGFP);neurog1*:*lRl-GFP*) embryos, in which *neurog1*:*lRl*(*loxP-DsRed-loxP*)*-GFP* transgenic line expresses only DsRed in the absence of Cre activity [35], *neurog1*:DsRed-positive neural progenitor cells underwent mitosis in the apical ventricular zone and these neural progenitor cells were clonally aligned with a couple of *neurod*:EGFP- and *neurog1*:DsRed-double-positive neurons in a column (S1B Fig). This suggests that neural progenitor cells that ultimately produce post-mitotic neurons undergo mitoses in the apical ventricular zone. These observations suggest that mitoses of neural progenitor cells in the sub-basal/sub-ventricular zone essentially produce post-mitotic neurons that are added apically onto the older neurons, while mitoses of neural progenitor cells in the ventricular zone contribute to amplification of neural progenitor cells to form clonal clusters of neural progenitor cells.

### ErbB signaling is implicated in the sub-ventricular mitoses

**Fig 2:**
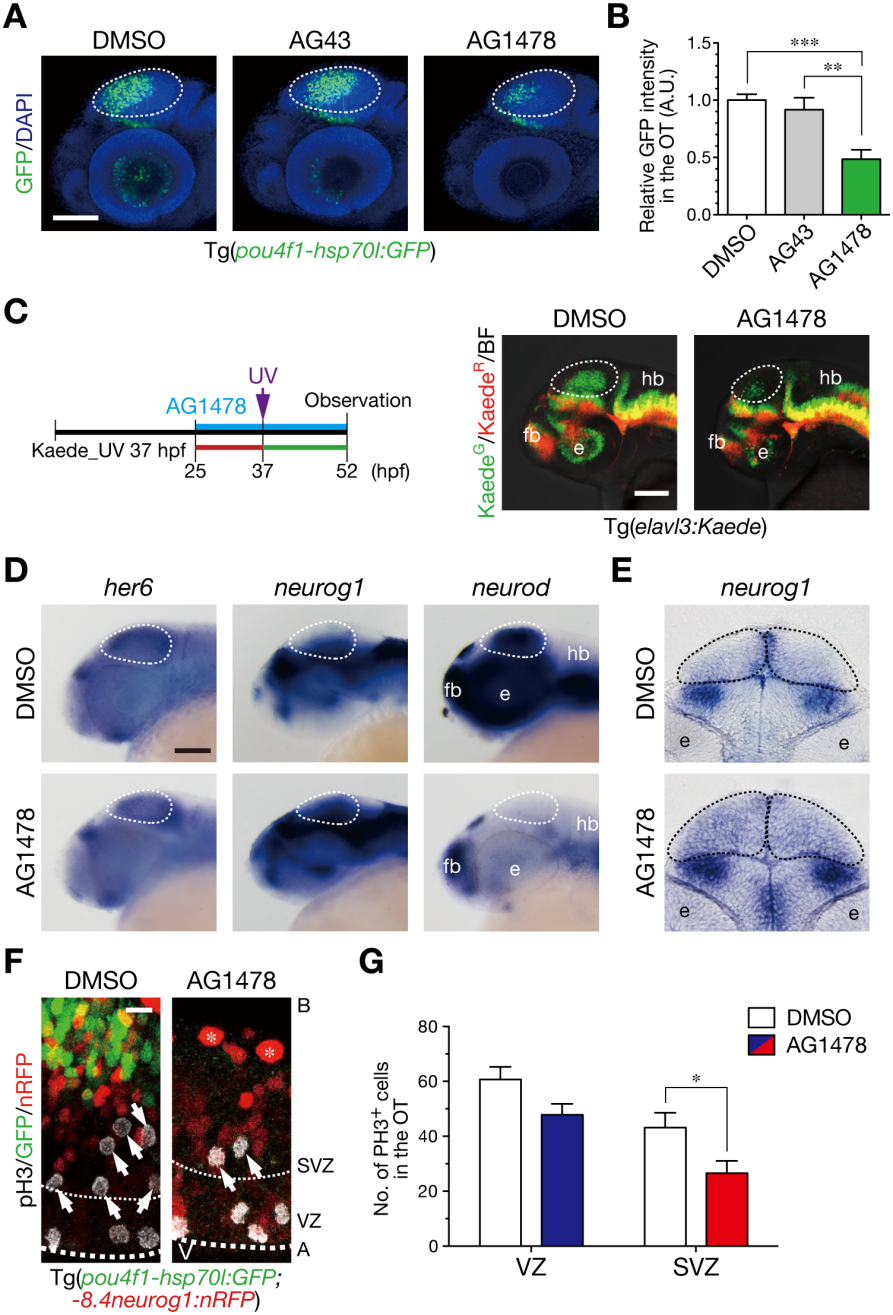
ErbB signaling is implicated in generation of neurons and mitoses in the sub-ventricular zone. **A**. Treatment with an ErbB inhibitor AG1478 prevents generation of GFP-expressing neurons in the OT of Tg(*pou4f1-hsp70l*:*GFP*) embryos, but not with the control AG43 and DMSO, at 52 hpf. Dotted circle, OT. Scale bar, 100 μm. **B**. Quantification of *pou4f1-hsp70l*:GFP intensity in the OT for the experiment shown in A (mean ± s.e.m.; **P < 0.01, ***P < 0.001; n = 8, 10, 16 for AG1478, AG43, DMSO, respectively). **C**. (left) A timeline of AG1478 treatment and irradiation of UV light on Tg(*elavl3*:*Kaede*). (right) Representative embryos showing impaired generation of neurons in the OT (dotted circle) at 52 hpf following AG1478 treatment. Scale bar, 50 μm. **D**. WISH of embryos at 44 hpf. Expression of *neurog1* and *neurod* is increased and diminished, respectively, while expression of *her6* is obviously unaltered in the OT following AG1478 treatment. Scale bar, 100 μm. **E**. A coronal section of WISH-stained embryos for *neurog1* mRNA at 36 hpf. **F**. Impaired neurogenesis and a decrease in pH3-positive mitotic cells in the SVZ of AG1478-treated Tg(*pou4f1-hsp70l*:*GFP;-8*.*4neurog1*:*nRFP*) embryos (AG1478) compared to the control embryos (DMSO) at 40 hpf. Scale bar, 10 μm. **G**. Quantification of the number of pH3-positive cells in the OT for the experiment shown in F, in the VZ (blue) and the SVZ (red) of AG1478-treated embryos and the control (white) embryos (mean ± s.e.m.; *P < 0.05; n = 11, 7 for AG1478, DMSO, respectively).

We next investigated whether these neurogenic cell divisions require NRG1-ErbB signaling, since NRG1-ErbB signaling is known to be a multi-potent regulator of neural development and functions in the nervous systems [8–10]. First, we examined the effects of an ErbB kinase inhibitor, AG1478, on neurogenesis in the optic tectum. When Tg(*pou4f1-hsp70l*:*GFP*) embryos were soaked in a solution of AG1478 from 26 hpf, they showed impaired generation of neurons in the optic tectum at 52 hpf, but not in a solution of ineffective derivative AG43 (Fig 2A and 2B). The effect of AG1478 on the generation of post-mitotic neurons was confirmed in TgBAC(*neurod*:*EGFP*) embryos (S2 Fig). UV irradiation on living Tg(*elavl3*:*Kaede*) embryos indicated the impairment of neuron generation, from the beginning of neurogenesis in the optic tectum (Fig 2C). Consistently, apoptosis was not induced in the optic tectum by the treatment with AG1478 (S3 Fig). Whole-mount *in situ* hybridization (WISH) showed that AG1478 treatment caused increased and expanded expression of *neurog1* and diminished expression of *neurod*, for markers of neural progenitor cells and post-mitotic neurons, respectively [7], while expression of *her6*, a *hes1* ortholog that is expressed in proliferating radial glial cells [42,43], was not obviously altered in the optic tectum (Fig 2D and 2E). Thus, the results implicate the effect of AG1478 not just in the developmental delay but in the arrested differentiation of neural progenitor cells into post-mitotic neurons. To examine effects of AG1478 on mitotic activity in the optic tectum, we quantified mitoses in the ventricular and sub-ventricular zones within the individual optic tectum of AG1478-treated and the control embryos. We found that cell divisions in the sub-ventricular zone were suppressed by the treatment with AG1478 at 40 hpf (Fig 2F and 2G). These results suggest that ErbB signaling is implicated in production of post-mitotic neurons and sub-ventricular mitoses in the optic tectum.

**Fig 3:**
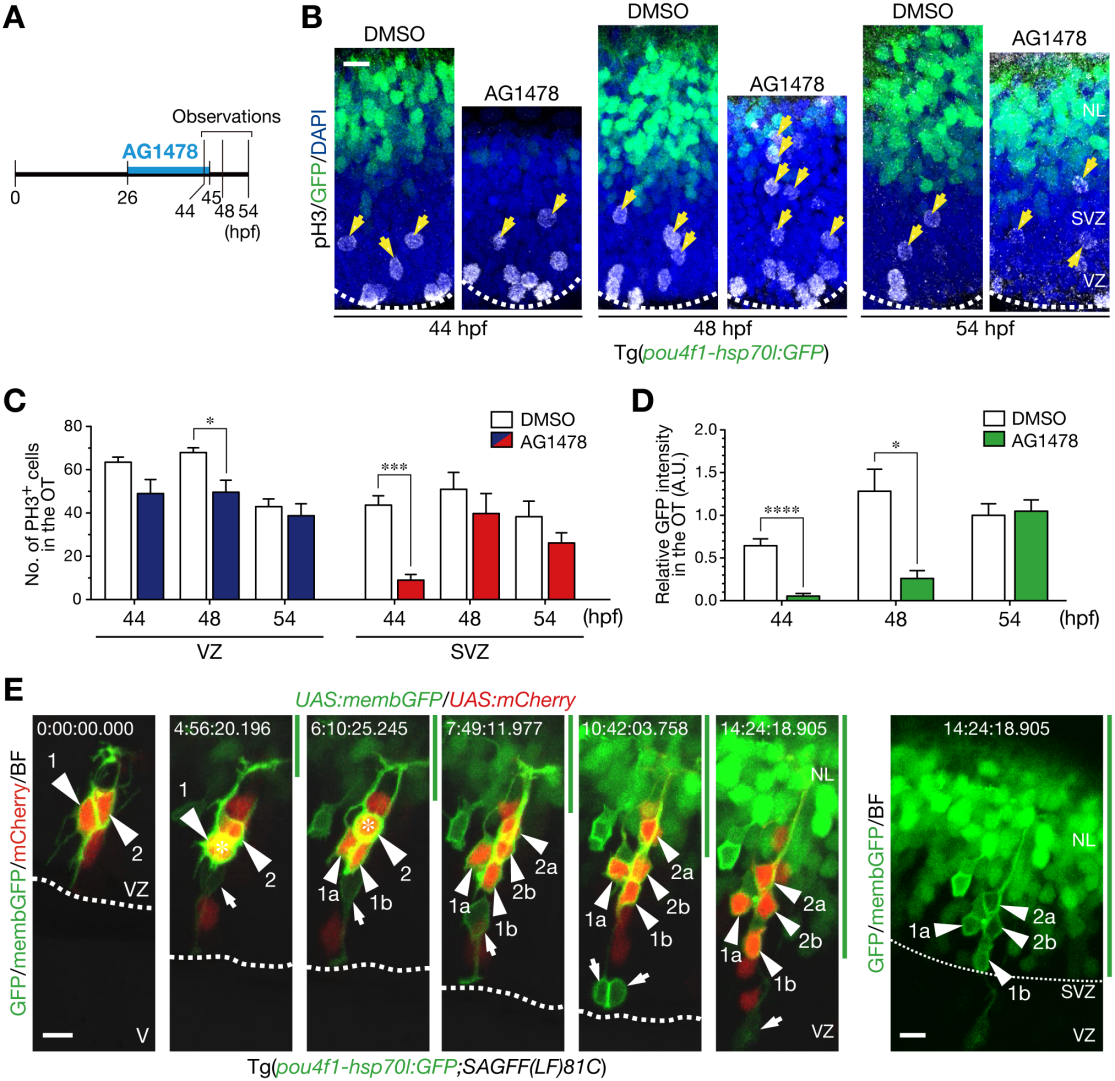
Mitoses generating neurons in the sub-ventricular zone require ErbB signaling. **A**. A timeline of experiments of AG1478 treatment. Embryos were soaked into 30 μM AG1478 solution or the control 2% DMSO from 26 to 45 hpf. Then, they were washed and grown in a fresh medium from 45 hpf. Embryos at 44, 48 and 54 hpf were collected for analyses. See also S3 Movie on the recovery of neurogenesis after removal of AG1478. **B**. pH3-positive mitotic cells in the OT. Removal of AG1478 at 45 hpf enhanced mitoses in the SVZ at 48 hpf prior to recovery of GFP-expressing neurons in the OT of Tg(*pou4f1-hsp70l*:*GFP*) at 54 hpf. Yellow arrows, pH3-positive cells in the SVZ. Scale bar, 10 μm. **C**. Quantification of the number of pH3-positive cells in the VZ (blue) and the SVZ (red) of AG1478-treated embryos and the control (white) embryos (mean ± s.e.m.; *P < 0.05, ***P < 0.001; n = 3, 5, 5 for AG1478, n = 6, 4, 6 for DMSO at 44, 48, 54 hpf, respectively). **D**. Quantification of GFP intensity in the OT (mean ± s.e.m.; *P < 0.05, ****P < 0.0001; n = 6, 4, 5 for AG1478, n = 6, 4, 6 for DMSO at 44, 48, 54 hpf, respectively). **E**. Time-lapse imaging of NPCs stochastically labeled by co-injection of *UAS*:*mCherry*/*UAS*:*memb*:*GFP* plasmids into Tg(*pou4f1-hsp70l*:*GFP*;*SAGFF(LF)81C*) embryos. After the removal of AG1478, two NPCs (1, 2; arrowheads) divided once in the SVZ (asterisk) to generate two daughter cells (a, b), and then, these cells were incorporated into the presumptive neuronal layer (NL, green bar, above the thin dotted line). Note that mitoses of these NPCs were not preceded by mitoses in the apical VZ and those NPCs began to express cytoplasmic GFP (right). An apical mitosis of another NPC or RGC labeled with membGFP was also observed in this imaging (arrow). Scale bar, 10 μm. See also S4 and S5 Movies.

The inhibitory effect of AG1478 on neurogenesis was reversible after removal of the inhibitor (S4 Fig). Live imaging of AG1478-treated embryos showed progression of basal-to-apical accumulation of neurons after removal of AG1478 (S3 Movie). Then, we examined whether neurogenic recovery accompanies initiation of mitoses in the ventricular zone or in the sub-ventricular zone after the removal of AG1478 in the protocol outlined in Fig 3A. It was confirmed that cell divisions in the sub-ventricular zone were also suppressed by the AG1478 treatment at 44 hpf (Fig 3B and 3C, 44 hpf). Following the removal of AG1478 at 45 hpf, cell divisions in the sub-ventricular zone were restored at 48 hpf without enhancing cell divisions in the apical region in Tg(*pou4f1-hsp70l*:*GFP*) embryos (Fig 3B and 3C, 48 hpf). The mitotic restoration occurred prior to the recovery of the number of differentiated neurons at 54 hpf, as evaluated by GFP intensity (Fig 3B and 3D). These results support an idea that ErbB signaling is directly involved in mitoses to generate post-mitotic neurons in the sub-ventricular zone. To investigate whether ErbB signaling directly regulates mitoses of sub-ventricular neural progenitor cells or indirectly affects them through activating mitoses of apical neural progenitor cells and/or radial glial cells, individual neurogenic cells were traced in living embryos after the removal of AG1478. We crossed Tg(*pou4f1-hsp70l*:*GFP*) with a gene-trap line SAGFF(LF)81C in which Gal4 is expressed in the neural stem/radial glial cells in the midbrain [36]. When the Tg(*pou4f1-hsp70l*:*GFP*;SAGFF(LF)81C) embryos were co-injected with *UAS*:*membGFP* and *UAS*:*mCherry*, single neural stem cell-derived cells were stochastically labeled with membGFP and mCherry individually. In AG1478-treated embryos, membGFP/mCherry double-positive cells in the sub-ventricular zone started to divide soon after the removal of AG1478 without undergoing precedent mitoses in the apical ventricular zone, and resultant daughter cells were incorporated into the *pou4f1-hsp70l*:GFP-positive neuronal layer (Fig 3E, S4 and S5 Movies), which is consistent with our observation that younger neurons were essentially located more apically to the layer of older neurons in the optic tectum of Tg(*elavl3*:*Kaede*) embryos (Fig 1E and 1F). The results indicate a direct role of ErbB signaling for stimulating neuron-producing mitoses of neural progenitor cells in the sub-ventricular zone.

### NRG1-II promotes mitoses in the ventricular and sub-ventricular zones

**Fig 4:**
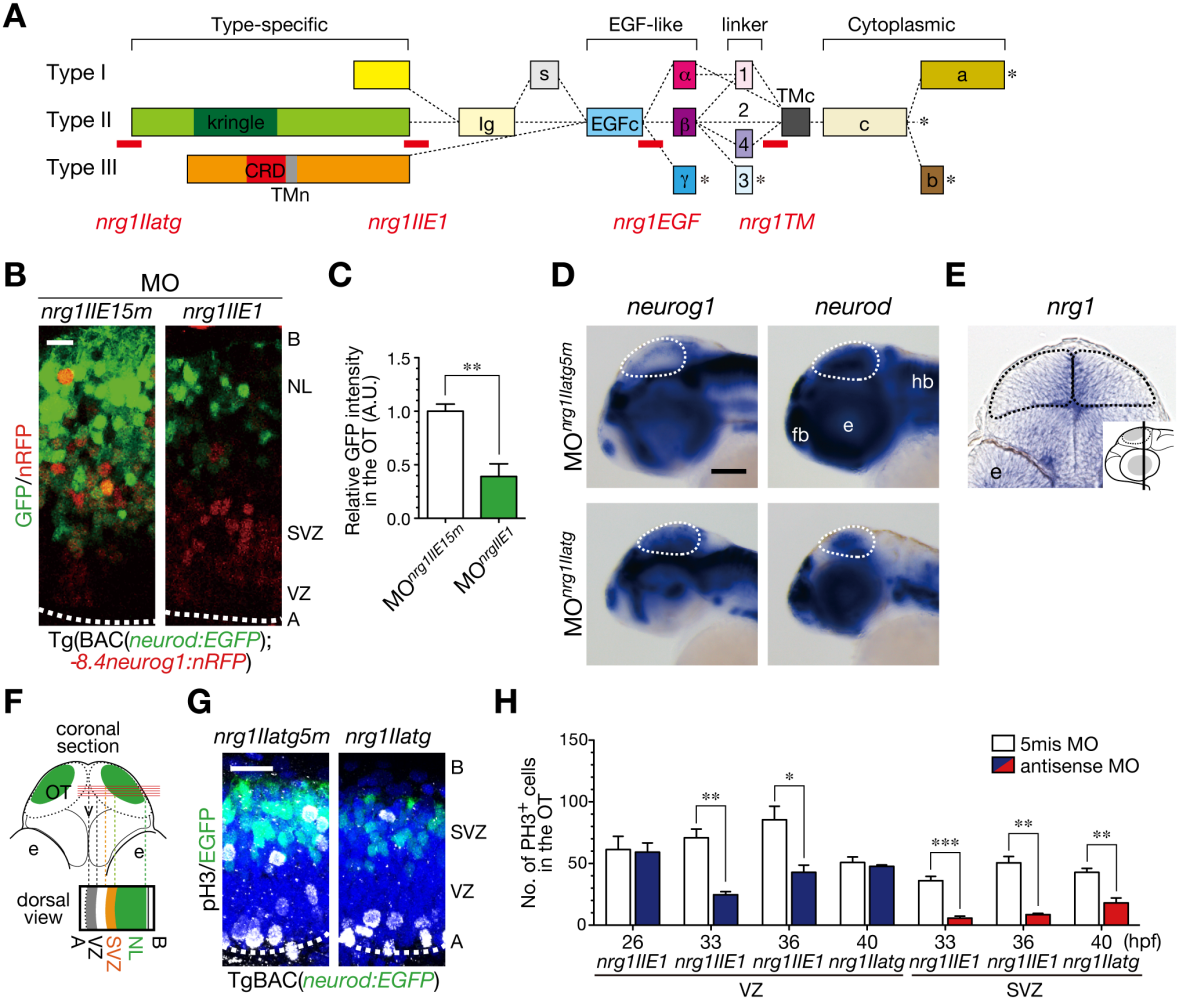
Generation of post-mitotic neurons requires NRG1-II. **A**. MOs against *nrg1* used in this study. Domain structures of NRG1 type I–III isoforms are shown together with the target site (red bar) of each *nrg1* MO. **B**. Decreased *neurod*:EGFP-positive neurons in MO^*nrg1IIE1*^-injected embryos (*nrg1IIE1*) compared to the control 5 nucleotides-mismatched MO^*nrg1IIE15m*^-injected embryos (*nrg1IIE15m*) of Tg(BAC(*neurod*:*GFP*);*-8*.*4neurog1*:*nRFP*) at 50 hpf. Scale bar, 10 μm. **C**. Quantification of *neurod*:EGFP intensity in the OT for the experiment shown in A (mean ± s.e.m.; **P < 0.01; n = 4 per group). **D**. WISH of MO^*nrg1IIatg*^-injected and the control MO^*nrg1IIatg5m*^-injected embryos for *neurog1* and *neurod* mRNAs at 48 hpf. Scale bar, 100 μm. **E**. A coronal section of WISH-stained embryos for *nrg1* mRNA at 36 hpf. The approximate site of the section is shown in a lateral view in the inset. **F**. A schematic of a stacked image of confocal sections from a dorsal view and the higher magnification of the OT from a dorsal view (bottom). **G**. Impaired neurogenesis and a decrease in pH3-positive mitotic cells in the SVZ of MO^*nrg1IIatg*^-injected TgBAC(*neurod*:*EGFP*) embryos (*nrg1IIatg*) compared to the control embryos (*nrg1IIatg5m*) at 40 hpf. Scale bar, 20 μm. **H**. Quantification of the number of pH3-positive cells in the VZ (blue) and the SVZ (red) of MO^*nrg1II*^-injected embryos and the control (white) embryos (mean ± s.e.m.; *P < 0.05, **P < 0.01, ***P < 0.001; n = 6, 6, 4, for *nrg1IIE1*, n = 6, 5, 5 for *nrg1IIE15m* at 26, 33, 36 hpf, n = 3, 4 for *nrg1IIatg*, *nrg1IIatg5m* at 40 hpf, respectively).

Because NRG1 has multiple isoforms by alternative splicing, diverse roles of NRG1-ErbB signaling are likely attributable to the multiple isoforms of NRG1 [8,9]. In zebrafish, at least three types of isoforms (type I–III) are reported for NRG1 according to the alternative splicing in the N-terminal domains [44]. To investigate whether NRG1 is involved in neurogenesis in the optic tectum, we introduced an antisense morpholino oligonucleotide (MO) against the EGF domain (MO^*nrg1EGF*^) shared by all the isoforms of NRG1 (Fig 4A) [37]. We found that knockdown of NRG1 caused defective generation of neurons in the optic tectum of Tg(*pou4f1-hsp70l*:*GFP*) embryos (S5A and S5B Fig). To elucidate which isoforms of NRG1 are involved in the sub-ventricular mitoses of neural progenitor cells, we injected MOs against type-I, II, and III (data not shown). When antisense MOs against NRG1-II (MO^*nrg1II*^; MO^*nrg1IIatg*^ and MO^*nrg1IIE1*^) were injected into Tg(*pou4f1-hsp70l*:*GFP*) or in Tg(BAC(*neurod*:*EGFP);-8*.*4neurog1*:*nRFP*) embryos, post-mitotic neurons were significantly decreased in the optic tectum, compared to those in embryos injected with the control MOs (MO^*nrg1IIatg5m*^ and MO^*nrg1IIE15m*^; Fig 4B and 4C and S5C and S5D Fig; phenotype ratios: *nrg1IIatg*, 0.79 ± 0.04, n = 104, *nrg1IIatg5m*, 0.05 ± 0.05, n = 115, *p* < 0.01; *nrg1IIE1*, 0.57 ± 0.10, n = 94, *nrg1IIE15m*, 0.02 ± 0.02, n = 36, *p* < 0.05), without increase in the number of apoptotic cells (S6 Fig). We confirmed that MO^*nrg1IIatg*^ specifically suppressed ectopic expression of NRG1-II from an expression plasmid *CMV*:*nrg1II-EGFP* (S7 Fig). WISH showed increased expression of *neurog1* and decreased expression of *neurod* in the optic tectum of MO^*nrg1II*^-injected embryos (Fig 4D), suggesting involvement of NRG1-II in production of post-mitotic neurons from neural progenitor cells.

Expression of *nrg1* was prominent in the apical region of the optic tectum (Fig 4E), suggesting production of NRG1 mainly by radial glial/neural progenitor cells in the ventricular zone. We therefore asked whether NRG1-II regulates either type of mitoses in the ventricular or sub-ventricular zone (Fig 4F–4H). In 26-hpf embryos, in which neural stem/radial glial cells are a predominant population, the majority of mitoses occurred exclusively in the apical ventricular zone, and there was no significant difference in the number of mitotic cells between MO^*nrg1II*^-injected and the control embryos (Fig 4H, 26 hpf). In contrast, the number of mitotic cells in MO^*nrg1II*^-injected embryos was significantly less than those in the controls at 33 and 36 hpf, both in the ventricular zone and in the sub-basal/sub-ventricular zone (Fig 4H, 33 and 36 hpf). Then at 40 hpf, while mitoses in the ventricular zone resumed, those in the sub-ventricular zone remained affected in MO^*nrg1II*^-injected embryos (Fig 4G and 4H, 40 hpf). These results suggest that NRG1-II is involved in the production of neurons by stimulating neurogenic mitoses of neural progenitor cells in the sub-ventricular zone and promoting their mitoses in the ventricular zone.

### Soluble human NRG1 rescues defective neurogenesis in NRG1-II-knockdown embryos

**Fig 5:**
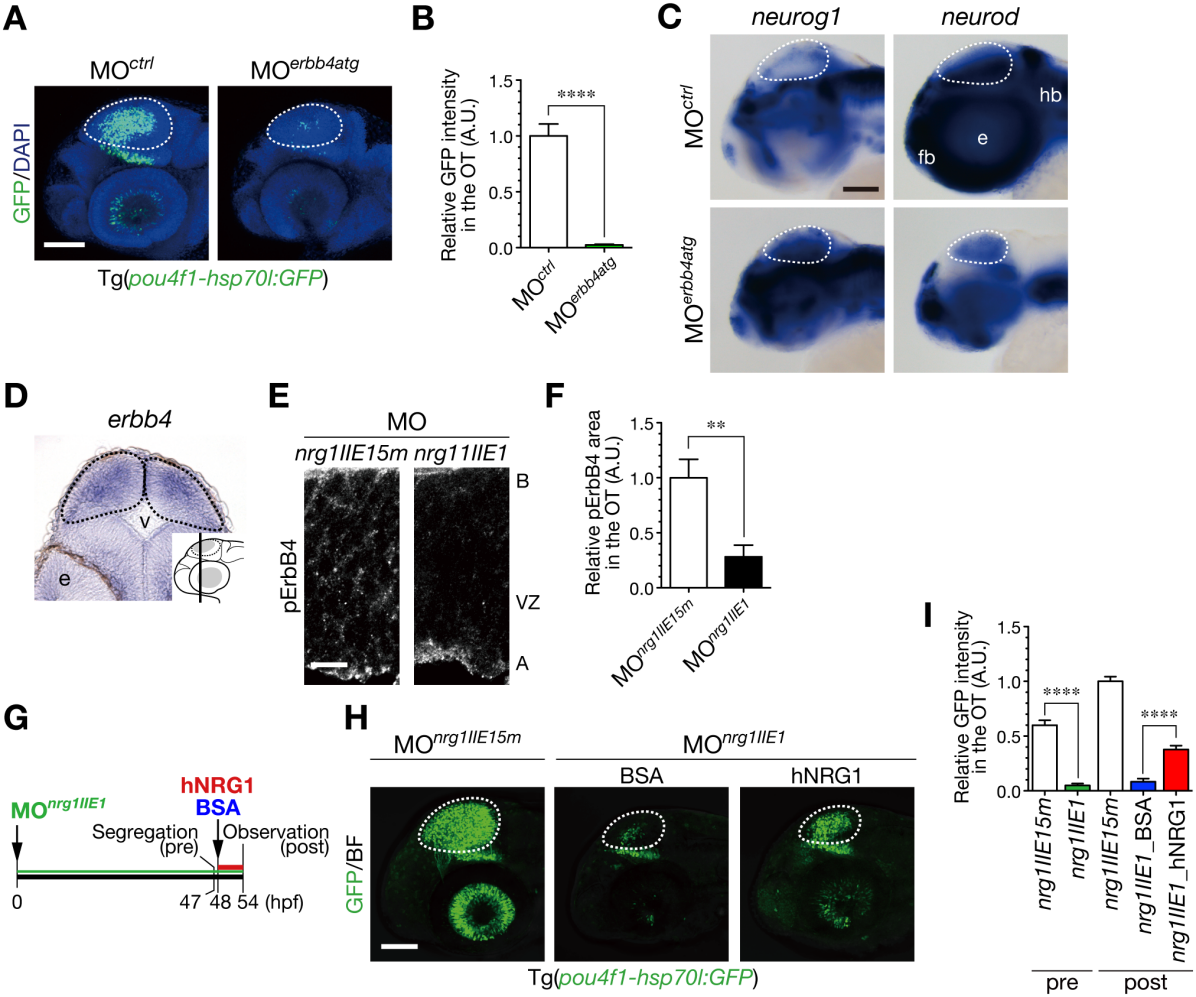
NRG1-II stimulates neurogenesis through ErbB4 as a cell-extrinsic signal. **A**. Impaired neurogenesis in MO^*erbb4atg*^-injected Tg(*pou4f1-hsp70l*:*GFP*) embryos at 50 hpf. Dotted circle, OT. Scale bar, 100 μm. **B**. Quantification of *pou4f1-hsp70l*:GFP intensity in the OT for the experiment shown in A (mean ± s.e.m.; ****P < 0.0001). **C**. WISH of MO^*erbb4atg*^-injected and the control MO^*ctrl*^-injected embryos for *neurog1* and *neurod* mRNAs at 48 hpf. Scale bar, 100 μm. **D**. A coronal section of WISH-stained embryos for *erbb4* mRNA at 36 hpf. The approximate site of the section is shown in a lateral view in the inset. **E**. A decrease in Tyr^1162^-phosphorylated ErbB4 (pErbB4) in the OT of MO^*nrg1IIE1*^-injected embryos (*nrg1IIE1*) compared to the control MO^*nrg1IIE15m*^-injected embryos (*nrg1IIE15m*) at 40 hpf. Scale bar, 10 μm. **F**. Quantification of pErbB4-positive area in the OT for the experiment shown in E (mean ± s.e.m; **P < 0.01; n = 8, 9 for MO^*nrg1IIE1*^, MO^*nrg1IIE15m*^, respectively). **G**. A timeline of the injection of hNRG1 proteins into the hindbrain ventricle. MO^*nrg1IIE1*^-injected embryos with neurogenic phenotypes were segregated at 47 hpf, and they were subjected to intra-ventricle injection of hNRG1 or the control BSA. These embryos were analyzed at 54 hpf. **H**. Partial rescue of the defective neurogenesis in MO^*nrg1IIE1*^-injected Tg(*pou4f1-hsp70l*:*GFP*) embryos at 54 hpf following the injection of hNRG1 proteins, compared to the control BSA injection. Scale bar, 100 μm. **I**. Quantification of *pou4f1-hsp70l*:GFP intensity in the OT for the experiment shown in H (mean ± s.e.m.; ****P < 0.0001; n = 17 for MO^*nrg1IIE1*^_pre, n = 21 for MO^*nrg1IIE15m*^_pre, n = 11 for MO^*nrg1IIE1*^_BSA, n = 16 for MO^*nrg1IIE1*^_hNRG1, n = 18 for MO^*nrg1IIE15m*^_post.).

To examine which ErbB receptor participates in neuronal generation in the developing optic tectum, we introduced antisense MOs against ErbB receptors into embryos of Tg(*pou4f1-hsp70l*:*GFP*) and found that an antisense MO against *erbb4* (MO^*erbb4*^; MO^*erbb4atg*^) impaired generation of post-mitotic neurons (Fig 5A and 5B; phenotype ratios: *erbb4atg*, 0.66 ± 0.05, n = 149, *Standard control*, 0.02 ± 0.01, n = 161, *p* < 0.01). Similar to the results with MO^*nrg1II*^-injected embryos, MO^*erbb4*^-injected embryos showed increased *neurog1* and decreased *neurod* expression in the optic tectum compared to the controls (Fig 5C), suggesting requirement of ErbB4 as a receptor for NRG1-II to stimulate differentiation of neural progenitor cells to post-mitotic neurons. While expression of *nrg1* was prominent in the apical region of the optic tectum (Fig 4E), *erbb4* was widely expressed in the optic tectum at 36 hpf (Fig 5D). These imply that NRG1 mainly produced by radial glial cells and/or neural progenitor cells in the ventricular zone stimulates ErbB4-expressing neural progenitor cells. Consistent with the expression of *erbb4* mRNA, Tyr^1162^-phosphorylated ErbB4 (pErbB4) was widely distributed in the optic tectum (S8 Fig). These pErbB4 signals significantly diminished in the optic tectum of MO^*nrg1II*^-injected embryos (Fig 5E and 5F), suggesting that NRG1-II stimulates neurogenesis by broadly activating ErbB signaling in the developing optic tectum. In general, membrane-bound EGF ligands are subject to proteolytic processing of their extracellular domains [8,9]. Injection of an antisense MO against an exon encoding the transmembrane (TM) region (MO^*nrg1TM*^; Fig 4A) also caused defective generation of neurons in the optic tectum (S5E and S5F Fig). These results imply a possibility that NRG1 ligands are produced as transmembrane proteins mainly in the ventricular zone, and then, activate ErbB4 to transduce neurogenic signals into neural progenitor cells after they are transported intracellularly and/or released by proteolytic processing. Indeed, defective production of neurons in MO^*nrg1II*^-injected embryos was partially restored by injection of recombinant soluble human NRG1 (hNRG1) proteins into the hindbrain ventricle (Fig 5G–5I). These results not only suggest the involvement of NRG1 as a cell-extrinsic signal for generation of neurons, but also imply a conserved role for NRG1-ErbB signaling in neurogenesis in the vertebrate brain from fish to humans.

## Discussion

In this study, we found that newborn neurons were essentially accumulated in the basal-to-apical direction, i.e. outside-in orientation, in the developing optic tectum. Time-lapse imaging *in vivo* revealed that the directional neuronal accumulation is closely linked to mitoses of sub-ventricular neural progenitor cells. After transient exposure of embryos to AG1478, mitoses in the sub-ventricular zone resumed without precedent cell divisions in the apical ventricular zone, suggesting that cell divisions of sub-ventricular neural progenitor cells *per se* are dependent on ErbB signaling. The exposure to AG1478 from 25 hpf could circumvent effects of the ErbB kinase inhibitor on early development, which allowed us to observe selective suppression of sub-ventricular cell divisions in AG1478-treated embryos. Actually, severe retardation in development was observed in embryos treated with AG1478 from an early stage of embryogenesis (data not shown). In contrast, because knockdown of NRG1-II not only suppressed cell divisions in the sub-basal/sub-ventricular zone but also affected those in the ventricular zone to some extent, proliferation and differentiation of neural progenitor cells likely require persistent exposure to the NRG1. Despite continuous suppression of cell divisions in the sub-basal/sub-ventricular zone in NRG1-II-knockdown embryos, the number of cell divisions in the ventricular zone was restored at 40 hpf, which implies a possibility that other ErbB ligands, such as other types of NRG1, NRG2, NRG3 and/or HB-EGF might be involved in the restoration. Membrane-bound isoforms of NRG1 are known to be subject to proteolytic processing within juxtamembrane domains encoded by exons α or β (ectodomain shedding) [8,9]. Injection of an antisense MO against the transmembrane domain of NRG1 caused inhibitory effects on neuron generation similar to isoform-specific MOs against NRG1-II, suggesting a requirement of a membrane-bound isoforms of NRG1-II for the neurogenesis. *nrg1* was predominantly expressed in the apical region of the optic tectum. Moreover, defective neurogenesis of NRG1-II-knockdown embryos was ameliorated by intra-ventricular injection of soluble human NRG1 proteins. These results imply the involvement of proteolytically processed, soluble NRG1 emanated from radial glial cells or neural progenitor cells in the ventricular zone for the long-range stimulation of neurogenesis.

In the optic tectum of zebrafish embryos, neural progenitor cells underwent mitoses in the ventricular zone and sub-basal/sub-ventricular zone, and the latter mitoses were involved in production of post-mitotic neurons. Mitotic neural progenitor cells in the two regions, ventricular and sub-ventricular zones, are also observed in the telencephalon and the hindbrain of mouse embryos as early as embryonic day 10.5, and most of neurons arise from basal progenitor cells in slice cultures of the embryonic telencephalon [39]. Thus, mitoses of neuron-generating neural progenitor cells likely occur in the sub-ventricular zone from the early stages of brain development, although it remains to be clarified whether accumulation of newborn neurons occurs in the basal-to-apical orientation also in the mammalian brain. A previous study shows that NRG1 type-I and -II isoforms are also expressed in the ventricular zone of the developing mouse brain [45], which is similar to the expression of *nrg1* in the apical region of the developing zebrafish optic tectum. Together with the significant rescue of neurogenesis in NRG1-II-knockdown zebrafish embryos by injecting human NRG1, the roles of NRG1 in production of neurons from the sub-ventricular neural progenitor cells would be conserved in the developing brain of vertebrates. Further investigations are needed to clarify whether NRG1-II plays critical roles in neurogenesis in the developing brain or whether functional redundancies exist among various isoforms of NRG1 or among NRG family proteins during brain development in mammals.

The balance between proliferation and differentiation of neural progenitor cells contributes to the size and shape of the brain [1,3]. Lineage tracing shows that daughter cells generated from radial glial cells divide once more to generate neurons in the forebrain or the hindbrain of zebrafish embryos [46,47]. In the developing optic tectum, the most expanded area in the zebrafish brain, neuron production in the sub-ventricular zone was preceded by amplification of neural progenitor cells in the ventricular zone, which is similar to the early phase of brain development in mammals [39]. By transient exposure to AG1478, cell divisions in the sub-ventricular zone were abrogated in the optic tectum. In addition, other brain areas, such as forebrain, tegmentum, eye, were presumably affected by the AG1478 treatment, which would result in small brains of treated embryos. More precise cell lineage analyses of neural progenitor cells and elucidation of molecular mechanisms regulating NRG1-II activity in the developing optic tectum and other brain areas will help us understand how mitotic balance of sub-ventricular and apical neural progenitor cells regulates the size and shape of the brain.

**Fig 6:**
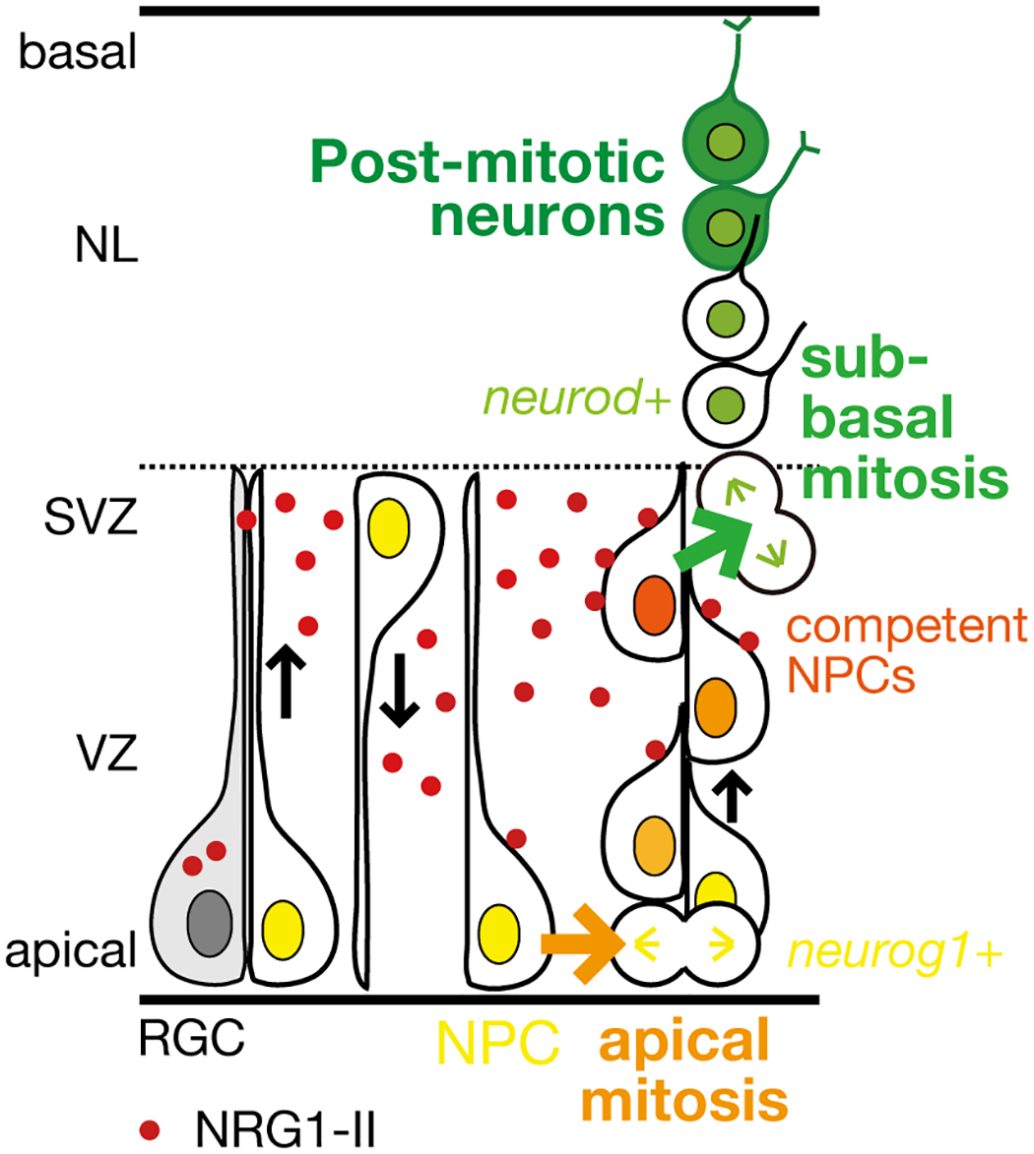
A schematic model for the roles of NRG1-II-ErbB signaling in neurogenesis in the optic tectum. NRG1-II (red dot) is produced from radial glial cells/neural progenitor cells (RGCs/NPCs) in the ventricular zone (VZ), and stimulates mitoses in the apical VZ (orange arrow), and also stimulates mitoses generating post-mitotic neurons in the sub-ventricular zone (SVZ; green arrow) by promoting neurogenic competence of NPCs.

Taken together, observation of neuron production in populations or in single cell levels using living zebrafish embryos allowed us to define roles of NRG1-ErbB in neurogenesis in the developing brain (Fig 6). Neural progenitor cells aligning in a row would receive NRG1 ligands, and divide to produce neurons one after another in the sub-ventricular zone in the basal to apical direction. The localized expression of *nrg1* mRNA in the apical region, which is in contrast to the dispersed distribution of phosphorylated ErbB4, lead us to propose a working model that radial glial cells and/or neural progenitor cells in the ventricular zone promote neurogenic competence of their daughter neural progenitor cells by secreting NRG1 from neural stem/radial glial cells with protrusions extended towards the basal region (Fig 6). Our finding on novel roles of NRG1-ErbB signaling in sub-ventricular neurogenesis in the developing brain not only would be noteworthy in order to understand a regulatory mechanism for terminal differentiation of neural progenitor cells, that is production of post-mitotic neurons from neural progenitor cells during brain development, but also might be useful for the etiologies of a psychiatric disorder with a defect in sensory-motor gating such as schizophrenia for which NRG1 and ErbB4 are linked as susceptibility loci.

## Supporting Information

S1 Fig Mitoses of neural progenitor cells in the apical ventricular zone generate clonaly aligned neural progenitor cells. **A**. Time-lapse imaging of neural progenitor cells (NPCs) in the optic tectum stochastically labeled by co-injection of *-8*.*4neurog1*:*Gal4VP16*/*UAS*:*memb*:*GFP/UAS*:*H2ARFP* plasmids into TgBAC(*neurod*:*EGFP*) embryos. NPC (3) underwent interkinetic nuclear migration prior to mitosis (asterisk) in the apical VZ. See also S2 Movie. A, apical; B, basal; NL, neuronal layer; VZ, ventricular zone. **B**. A single cell lineage of a TgBAC(*neurod*:*EGFP;neurog1*:*lRl*(*loxP-DsRed-loxP*)*-GFP*) embryo including both *neurod*:EGFP-positive neurons (arrows) and a dividing *neurog1*:DsRed-positive NPC (arrow head). TgBAC(*neurog1*:*lRl-GFP*) expresses DsRed in the absence of Cre.
(TIF)

S2 Fig Generation of post-mitotic neurons is impaired in the optic tectum by treatment with an ErbB inhibitor AG1478. **A**. Impaired generation of post-mitotic neurons in the optic tectum (OT; dotted circle) at 48 hpf following AG1478 treatment in TgBAC(*neurod*:*EGFP*) embryos, shown in a lateral view. Scale bar, 100 μm. **B**. Decreased *neurod*:EGFP-positive post-mitotic neurons and accumulated *-8*.*4neurog1*:nRFP-positive NPCs at 48 hpf by AG1478 treatment. A, apical; B, basal; NL, neuronal layer; SVZ, sub-ventriular zone; VZ, ventricular zone. Scale bar, 10 μm. **C**. Quantification of *neurod*:EGFP intensity in the OT for the experiment shown in A (mean ± s.e.m.; *P < 0.05, unpaired t test; n = 4–5).
(TIF)

S3 Fig Treatment with AG1478 does not induce apoptosis in the optic tectum. **A**. Immunohistochemical staining of embryos treated with AG1478 and the control DMSO with anti-activated Caspase-3 antibody at 38 hpf. Arrow, Caspase-3-positive puncta; dotted circle, optic tectum (OT). **B**. Quantification of the number of Caspase-3-positive puncta in the OT for the experiment shown in A (mean ± s.e.m.; DMSO, 2.4 ± 0.93, n = 5, AG1478, 3.0 ± 1.00, n = 5, *p* = 0.67, unpaired t test).
(TIF)

S4 Fig Generation of GFP-expressing neurons in the optic tectum is partially recovered after removal of AG1478. **A**. A timeline of experiments of AG1478 treatment. Embryos were soaked into 25 μM AG1478 solution or the control DMSO from 25 to 50 hpf. Then, they were washed and grown in a fresh medium. Embryos at 72 hpf were collected for analyses. **B**. Decreased *pou4f1-hsp70l*:GFP-positive neurons in the optic tectum of AG1478-treated embryos at 50 hpf were partially recovered at 72 hpf compared to the control DMSO in Tg(*pou4f1-hsp70l*:*GFP*).
(TIF)

S5 Fig Defective generation of GFP-expressing neurons in the optic tectum by injection of antisense MOs against *nrg1*. **A**. Knockdown of all isoforms of NRG1 by injection of MO^*nrg1EGF*^. Representative embryos injected with MO^*nrg1EGF*^ (right) or the control MO^*ctrl*^ (left) shown in a lateral view at 48 hpf. Dotted circle, optic tectum (OT). Scale bar, 100 μm. **B**. Quantification of *pou4fl-hsp70l*:GFP intensity in the OT for the experiment shown in A (mean ± s.e.m.; ****P < 0.0001, unpaired t test; n = 6–7). **C**. Impaired neurogenesis in a representative MO^*nrg1IIE1*^-injected Tg(*pou4f1-hsp70l*:*GFP*) embryo (right) compared to the control MO^*nrg1IIE15m*^ (5 nucleotides-mismatched control)-injected embryo (left) at 50 hpf. Scale bar, 100 μm. **D**. Quantification of *pou4f1-hsp70l*:GFP intensity in the OT for the experiment shown in C (mean ± s.e.m.; ****P < 0.0001; n = 8 per group). **E**. Knockdown of membrane-bound isoforms of NRG1 by injection of MO^*nrg1TM*^. Representative embryos injected with MO^*nrg1TM*^ (right) or the control, MO^*nrg1TM5m*^ (left) shown in a lateral view at 53 hpf. Dotted circle, OT. Scale bar, 100 μm. **F**. Quantification of *pou4f1-hsp70l*:GFP intensity in the OT for the experiment shown in E (mean ± s.e.m.; ****P < 0.0001, unpaired t test; n = 6 per group).
(TIF)

S6 Fig Injection of MO^*nrg1IIE1*^ does not induce apoptosis in the optic tectum. **A**. Immunohistochemical staining of embryos injected with MO^*nrg1IIE1*^ and the control MO^*nrg1IIE15m*^ with anti-activated Caspase-3 antibody at 50 hpf. Higher magnification of the Caspase-3-positive punctum is shown in the inset. Arrow, Caspase-3-positive puncta; dotted circle, optic tectum (OT). **B**. Quantification of the number of Caspase-3-positive puncta in the OT for the experiment shown in A (mean ± s.e.m.; *nrg1IIE15m*, 6.2 ± 3.54, n = 5, *nrg1IIE1*, 5.0 ± 2.05, n = 5, *p* = 0.78, unpaired t test).
(TIF)

S7 Fig MO^*nrg1IIatg*^ specifically suppresses ectopic expression of NRG1-II. **A**. A schematic structure of an expression plasmid *CMV*:*nrg1II-EGFP* (top), a part of the nucleotide and amino acid sequences encoding 5’ untranslated and coding regions in the first exon of *nrg1-II* (middle), and the target sequence of MO^*nrg1IIatg*^ (bottom, red arrow). **B**. Representative 74-hpf embryos co-injected with *CMV*:*nrg1II-EGFP* expression plasmid and MO^*nrg1IIatg*^ or the control MO^*nrg1IIatg5m*^ shown in a lateral view. Green fluorescence in yolk of MO^*nrg1IIatg*^-injected embryos is autoflurescence (arrowhead). Scale bar, 500 μm. **C**. Quantification of ratios of GFP-positive embryos co-injected with *CMV*:*nrg1II-EGFP* and MO^*nrg1IIatg*^ or the control MO^*nrg1IIatg5m*^ at 25 hpf. No GFP-positive embryos were detected for MO^*nrg1IIatg*^-injected embryos under a fluorescent dissection microscopy. (mean ± s.e.m.; *nrg1IIatg5m*, 0.56 ± 0.08, n = 183 embryos by 4 injections, *nrg1IIatg*, 0.00 ± 0.00, n = 98 embryos by 4 injections).
(TIF)

S8 Fig Immunostained signals for Tyr^1162^-phosphorylated ErbB4 are diminished in the optic tectum by injection of MO^*erbb4atg*^. **A**. Alignment of amino acid sequences of human ERBB4 and zebrafish ErbB4 around Tyr^1188^ of human ERBB4. Identical amino acids are indicated by shaded boxes. A tyrosine residue recognized by the anti-phospho-HER4 pTyr1188 (pErbB4) antibody is shown in red. **B**. Immunohistochemical staining of cryosections of Tg(*pou4f1-hsp70l*:*GFP*) embryos at 48 hpf with the anti-pErbB4 antibody. Embryos were injected with MO^*erbb4atg*^ (right) or with standard control MO^*ctrl*^ (left). Yellow dotted circle, optic tectum (OT); e, eye; v, ventricle. Images at higher magnification in the OT are shown below. The strong signals for pErbB4 in the basal region (red arrows) would be probably derived from ErbB4 localized in dendrites of neurons, because the signals are disappeared in embryos injected with MO^*erbb4atg*^. A, apical; B, basal. Scale bars, 50 μm (top), 10μm (bottom). **C**. Quantification of volume of pErbB4 puncta in the OT for the experiment shown in B (mean ± s.e.m.; **P < 0.01, unpaired t test; n = 7–8). The strong signals in the basal region (B, red arrows) are not included in this analysis.
(TIF)

S1 Movie Mitoses of neural progenitor cells in the sub-ventricular zone generate post-mitotic neurons. Time-lapse imaging of neural progenitor cells (NPCs) stochastically labeled by co-injection of *-8*.*4neurog1*:*Gal4VP16*/ *UAS*:*memb*:*GFP*/*UAS*:*H2ARFP* plasmids into TgBAC(*neurod*:*EGFP*) embryos. Two NPCs (1, 2) undergo mitoses in the sub-ventricular zone (SVZ; asterisk) to produce two daughter cells (a, b) that ultimately differentiate into *neurod*:GFP-expressing post-mitotic neurons in the neuronal layer (1a, 1b, 2a).
(MOV)

S2 Movie Mitoses of neural progenitor cells in the apical ventricular zone generate clonally aligned neural progenitor cells. Time-lapse imaging of another neural progenitor cell (3) that undergoes interkinetic nuclear migration prior to mitosis (asterisk) in the apical ventricular zone.
(MOV)

S3 Movie Neurogenesis is recovered after removal of AG1478. Live imaging of AG1478-treated embryos showing progression of basal-to-apical accumulation of neurons after removal of AG1478. V, ventricle; VZ, ventricular zone; SVZ, sub-ventricular zone; asterisk, pigment cell in the skin.
(MOV)

S4 Movie Time-lapse imaging of neural progenitor cells after removal of AG1478. Neural progenitor cells (NPCs) were stochastically labelled by co-injection of *UAS*:*mCherry*/*UAS*:*memb*:*GFP* plasmids into Tg(*pou4f1-hsp70l*:*GFP*;*SAGFF(LF)81C*) embryos. After the removal of AG1478 (50 hpf), two NPCs (1, 2) started to divide once in the sub-ventricular zone (SVZ; asterisk) to generate two daughter cells (a, b), and then, these cells were incorporated into the neuronal layer (NL, above the dotted line). Note that mitoses of these NPCs are not preceded by mitoses in the apical ventricular zone (VZ). An apical mitosis of another NPC or radial glial cell labeled with membGFP was also observed in the background (arrow).
(MOV)

S5 Movie Time-lapse imaging of neural progenitor cells after removal of AG1478. Neural progenitor cells (NPCs) were stochastically labelled by co-injection of *UAS*:*mCherry*/*UAS*:*memb*:*GFP* plasmids into Tg(*pou4f1-hsp70l*:*GFP*;*SAGFF(LF)81C*) embryos. After the removal of AG1478 (48 hpf), two NPCs (1, 2) started to divide once in the sub-ventricular zone (SVZ; asterisk) to generate two daughter cells (a, b), and then, three cells (1a, 1b, 2b) were incorporated into the neuronal layer (NL, above the dotted line). Note that mitosis of another NPC in the SVZ (small asterisk) generates daughter cells that remain in the SVZ (arrows).
(MOV)
