# Supplementary figures and images for: Neuregulin 1 Type II-ErbB Signaling Promotes Cell Divisions Generating Neurons from Neural Progenitor Cells in the Developing Zebrafish Brain
Source: PLoS One. 2015 May 22;10(5):e0127360. doi: 10.1371/journal.pone.0127360 (PMC4441363; doi:10.1371/journal.pone.0127360)

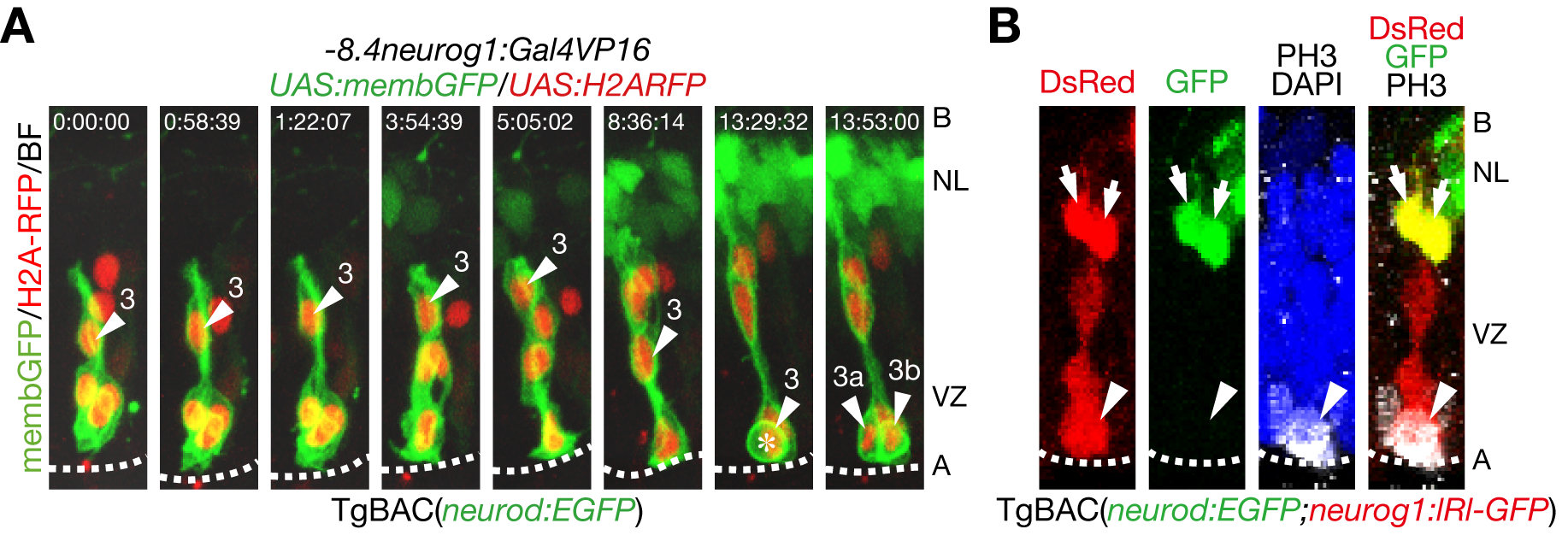

Supplement: S1 Fig — A. Time-lapse imaging of neural progenitor cells (NPCs) in the optic tectum stochastically labeled by co-injection of -8.4neurog1:Gal4VP16/UAS:memb:GFP/UAS:H2ARFP plasmids into TgBAC(neurod:EGFP) embryos. NPC (3) underwent interkinetic nuclear migration prior to mitosis (asterisk) in the apical VZ. See also S2 Movie. A, apical; B, basal; NL, neuronal layer; VZ, ventricular zone. B. A single cell lineage of a TgBAC(neurod:EGFP;neurog1:lRl(loxP-DsRed-loxP)-GFP) embryo including both neurod:EGFP-positive neurons (arrows) and a dividing neurog1:DsRed-positive NPC (arrow head). TgBAC(neurog1:lRl-GFP) expresses DsRed in the absence of Cre. (TIF) [file pone.0127360.s001.tif]

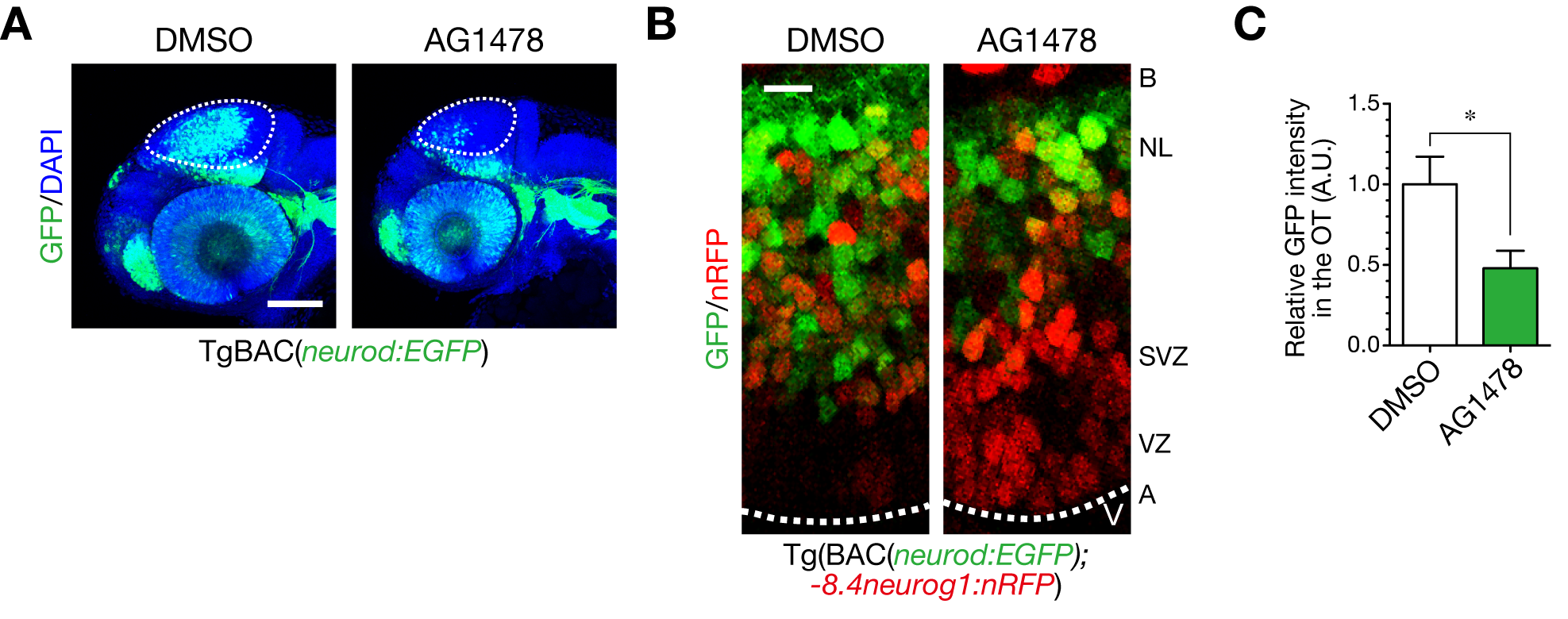

Supplement: S2 Fig — A. Impaired generation of post-mitotic neurons in the optic tectum (OT; dotted circle) at 48 hpf following AG1478 treatment in TgBAC(neurod:EGFP) embryos, shown in a lateral view. Scale bar, 100 μm. B. Decreased neurod:EGFP-positive post-mitotic neurons and accumulated -8.4neurog1:nRFP-positive NPCs at 48 hpf by AG1478 treatment. A, apical; B, basal; NL, neuronal layer; SVZ, sub-ventriular zone; VZ, ventricular zone. Scale bar, 10 μm. C. Quantification of neurod:EGFP intensity in the OT for the experiment shown in A (mean ± s.e.m.; *P < 0.05, unpaired t test; n = 4–5). (TIF) [file pone.0127360.s002.tif]

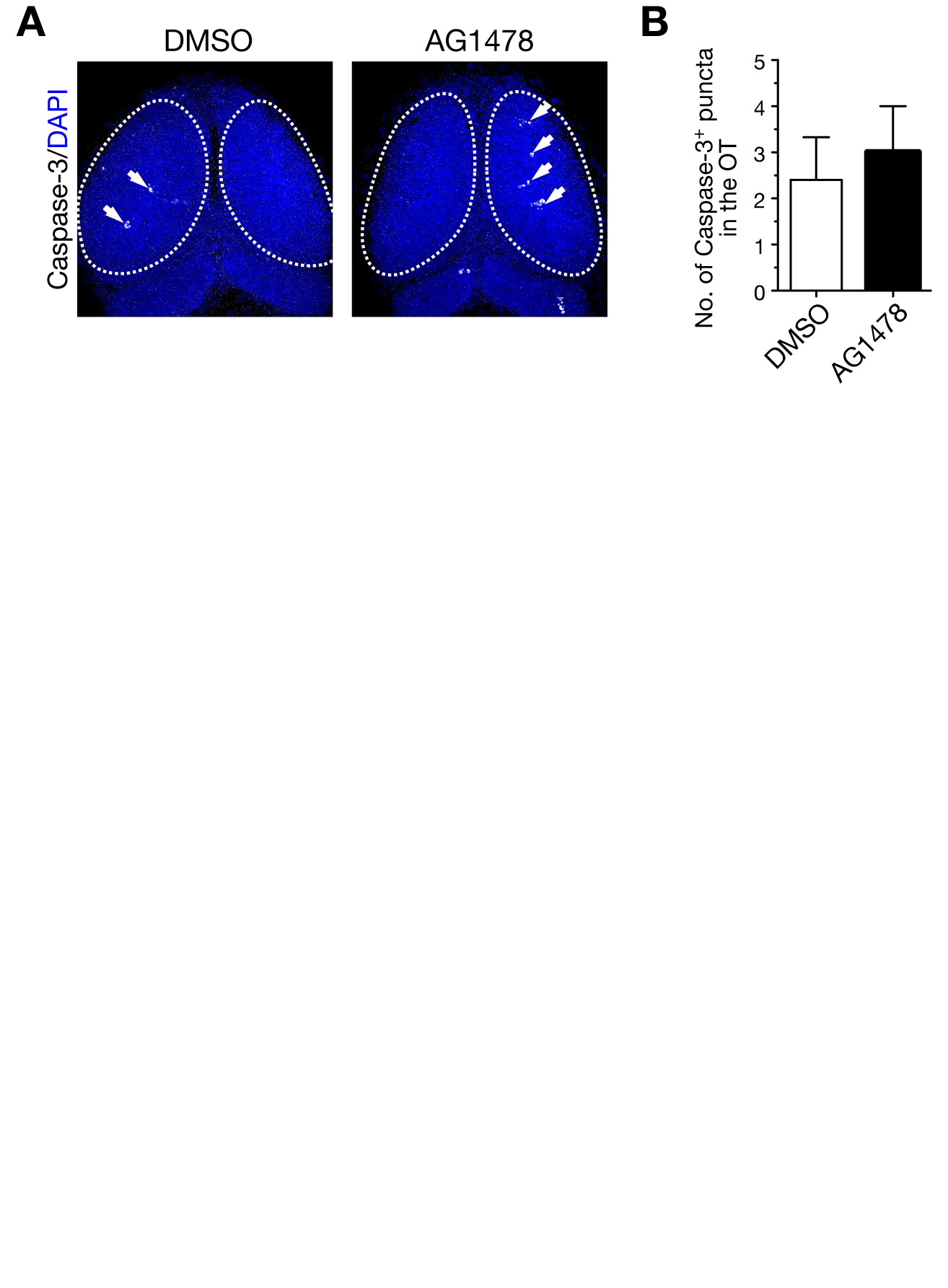

Supplement: S3 Fig — A. Immunohistochemical staining of embryos treated with AG1478 and the control DMSO with anti-activated Caspase-3 antibody at 38 hpf. Arrow, Caspase-3-positive puncta; dotted circle, optic tectum (OT). B. Quantification of the number of Caspase-3-positive puncta in the OT for the experiment shown in A (mean ± s.e.m.; DMSO, 2.4 ± 0.93, n = 5, AG1478, 3.0 ± 1.00, n = 5, p = 0.67, unpaired t test). (TIF) [file pone.0127360.s003.tif]

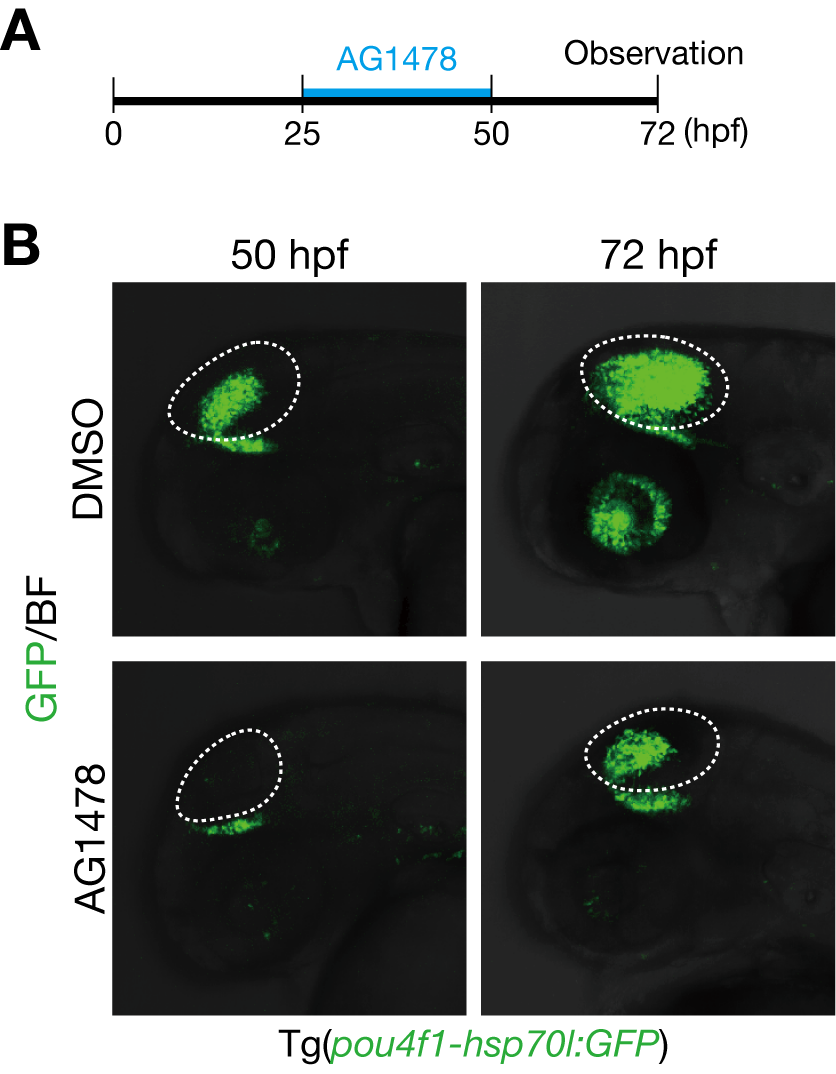

Supplement: S4 Fig — A. A timeline of experiments of AG1478 treatment. Embryos were soaked into 25 μM AG1478 solution or the control DMSO from 25 to 50 hpf. Then, they were washed and grown in a fresh medium. Embryos at 72 hpf were collected for analyses. B. Decreased pou4f1-hsp70l:GFP-positive neurons in the optic tectum of AG1478-treated embryos at 50 hpf were partially recovered at 72 hpf compared to the control DMSO in Tg(pou4f1-hsp70l:GFP). (TIF) [file pone.0127360.s004.tif]

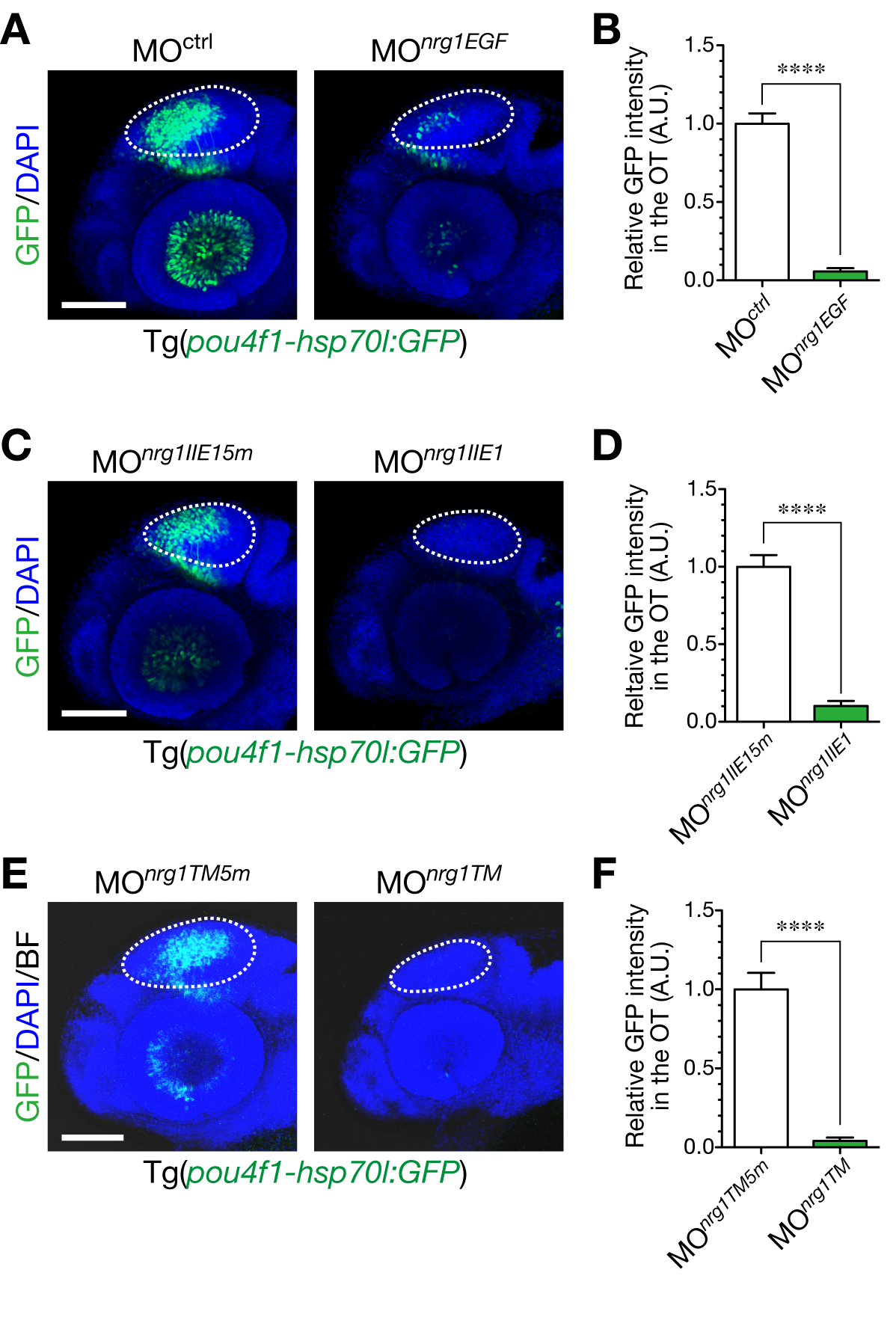

Supplement: S5 Fig — A. Knockdown of all isoforms of NRG1 by injection of MOnrg1EGF. Representative embryos injected with MOnrg1EGF (right) or the control MOctrl (left) shown in a lateral view at 48 hpf. Dotted circle, optic tectum (OT). Scale bar, 100 μm. B. Quantification of pou4fl-hsp70l:GFP intensity in the OT for the experiment shown in A (mean ± s.e.m.; ****P < 0.0001, unpaired t test; n = 6–7). C. Impaired neurogenesis in a representative MOnrg1IIE1-injected Tg(pou4f1-hsp70l:GFP) embryo (right) compared to the control MOnrg1IIE15m (5 nucleotides-mismatched control)-injected embryo (left) at 50 hpf. Scale bar, 100 μm. D. Quantification of pou4f1-hsp70l:GFP intensity in the OT for the experiment shown in C (mean ± s.e.m.; ****P < 0.0001; n = 8 per group). E. Knockdown of membrane-bound isoforms of NRG1 by injection of MOnrg1TM. Representative embryos injected with MOnrg1TM (right) or the control, MOnrg1TM5m (left) shown in a lateral view at 53 hpf. Dotted circle, OT. Scale bar, 100 μm. F. Quantification of pou4f1-hsp70l:GFP intensity in the OT for the experiment shown in E (mean ± s.e.m.; ****P < 0.0001, unpaired t test; n = 6 per group). (TIF) [file pone.0127360.s005.tif]

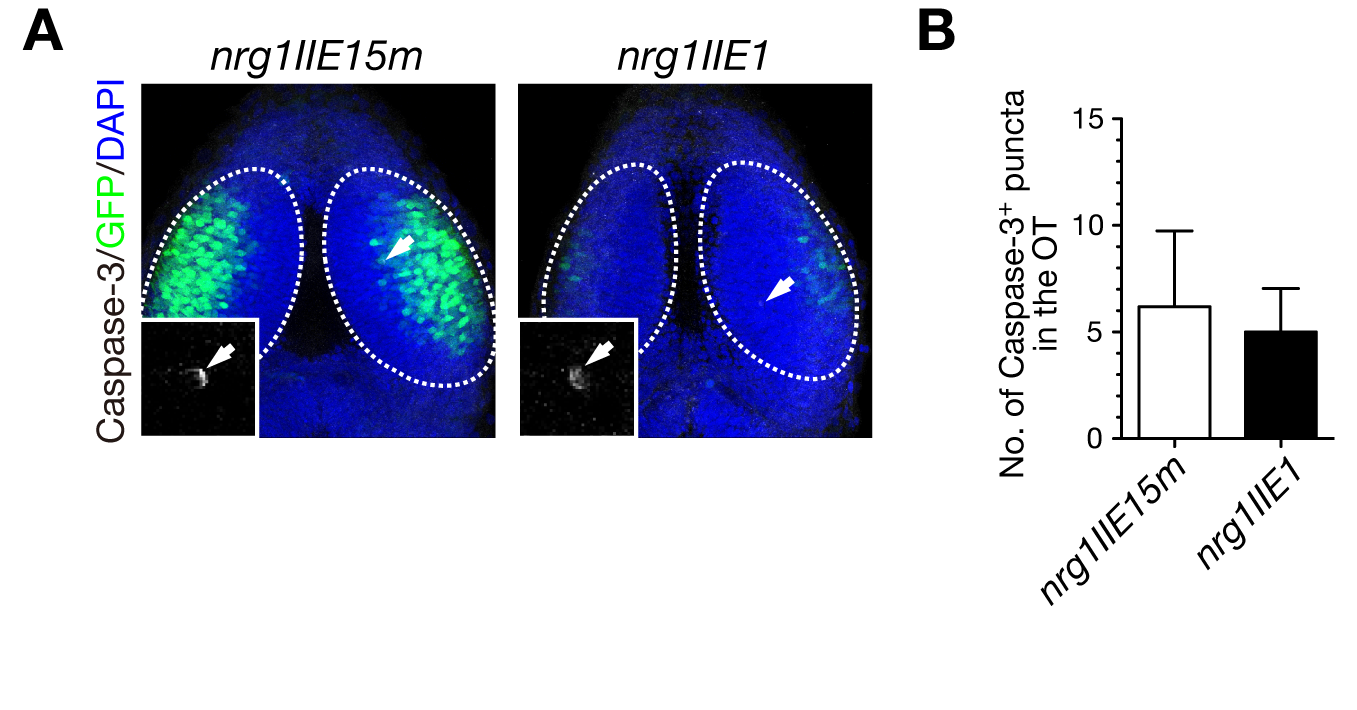

Supplement: S6 Fig — A. Immunohistochemical staining of embryos injected with MOnrg1IIE1 and the control MOnrg1IIE15m with anti-activated Caspase-3 antibody at 50 hpf. Higher magnification of the Caspase-3-positive punctum is shown in the inset. Arrow, Caspase-3-positive puncta; dotted circle, optic tectum (OT). B. Quantification of the number of Caspase-3-positive puncta in the OT for the experiment shown in A (mean ± s.e.m.; nrg1IIE15m, 6.2 ± 3.54, n = 5, nrg1IIE1, 5.0 ± 2.05, n = 5, p = 0.78, unpaired t test). (TIF) [file pone.0127360.s006.tif]

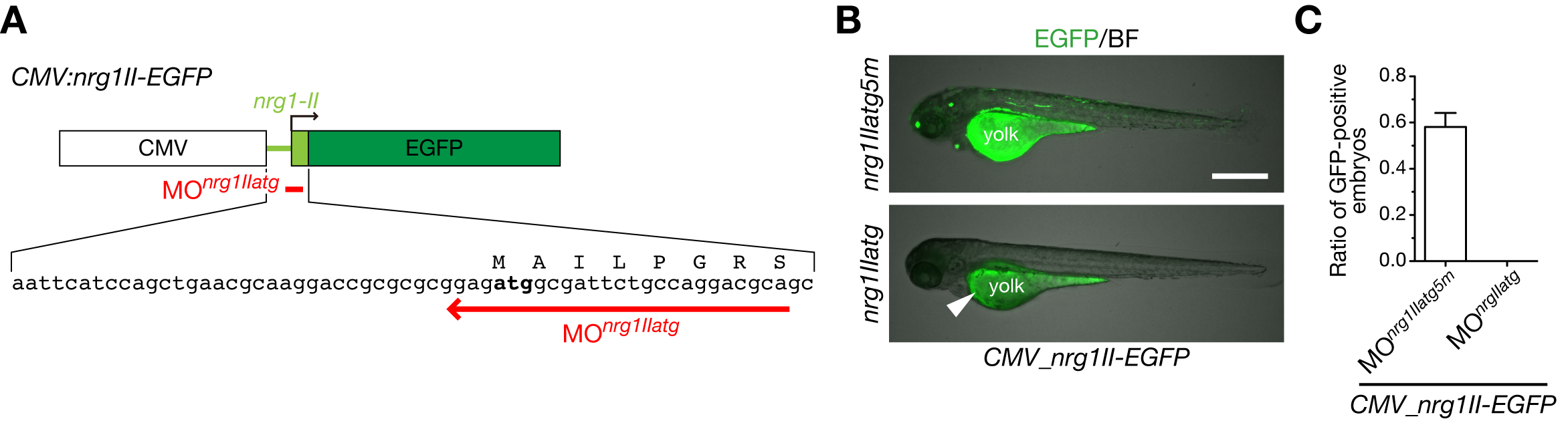

Supplement: S7 Fig — A. A schematic structure of an expression plasmid CMV:nrg1II-EGFP (top), a part of the nucleotide and amino acid sequences encoding 5’ untranslated and coding regions in the first exon of nrg1-II (middle), and the target sequence of MOnrg1IIatg (bottom, red arrow). B. Representative 74-hpf embryos co-injected with CMV:nrg1II-EGFP expression plasmid and MOnrg1IIatg or the control MOnrg1IIatg5m shown in a lateral view. Green fluorescence in yolk of MOnrg1IIatg-injected embryos is autoflurescence (arrowhead). Scale bar, 500 μm. C. Quantification of ratios of GFP-positive embryos co-injected with CMV:nrg1II-EGFP and MOnrg1IIatg or the control MOnrg1IIatg5m at 25 hpf. No GFP-positive embryos were detected for MOnrg1IIatg-injected embryos under a fluorescent dissection microscopy. (mean ± s.e.m.; nrg1IIatg5m, 0.56 ± 0.08, n = 183 embryos by 4 injections, nrg1IIatg, 0.00 ± 0.00, n = 98 embryos by 4 injections). (TIF) [file pone.0127360.s007.tif]

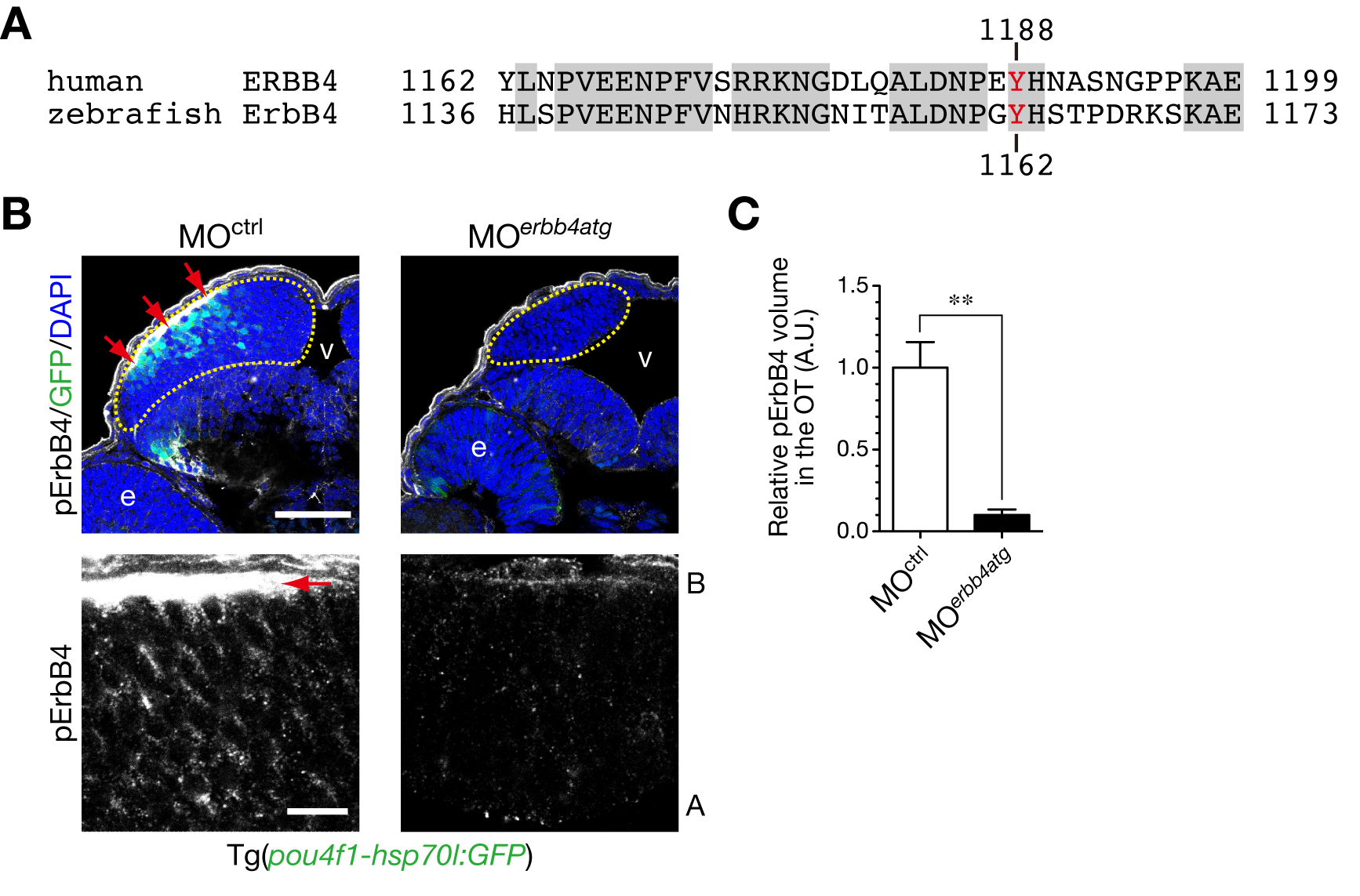

Supplement: S8 Fig — A. Alignment of amino acid sequences of human ERBB4 and zebrafish ErbB4 around Tyr1188 of human ERBB4. Identical amino acids are indicated by shaded boxes. A tyrosine residue recognized by the anti-phospho-HER4 pTyr1188 (pErbB4) antibody is shown in red. B. Immunohistochemical staining of cryosections of Tg(pou4f1-hsp70l:GFP) embryos at 48 hpf with the anti-pErbB4 antibody. Embryos were injected with MOerbb4atg (right) or with standard control MOctrl (left). Yellow dotted circle, optic tectum (OT); e, eye; v, ventricle. Images at higher magnification in the OT are shown below. The strong signals for pErbB4 in the basal region (red arrows) would be probably derived from ErbB4 localized in dendrites of neurons, because the signals are disappeared in embryos injected with MOerbb4atg. A, apical; B, basal. Scale bars, 50 μm (top), 10μm (bottom). C. Quantification of volume of pErbB4 puncta in the OT for the experiment shown in B (mean ± s.e.m.; **P < 0.01, unpaired t test; n = 7–8). The strong signals in the basal region (B, red arrows) are not included in this analysis. (TIF) [file pone.0127360.s008.tif]
